# Comparative Transcriptomics Atlases Reveals Different Gene Expression Pattern Related to *Fusarium* Wilt Disease Resistance and Susceptibility in Two *Vernicia* Species

**DOI:** 10.3389/fpls.2016.01974

**Published:** 2016-12-27

**Authors:** Yicun Chen, Hengfu Yin, Ming Gao, Huiping Zhu, Qiyan Zhang, Yangdong Wang

**Affiliations:** ^1^State Key Laboratory of Tree Genetics and Breeding, Chinese Academy of ForestryBejing, China; ^2^Institute of Subtropical Forestry, Chinese Academy of ForestryHangzhou, China

**Keywords:** *Vernicia* species, *Fusarium* wilt, comparative transcriptomics, resistance genes, co-expression

## Abstract

*Vernicia fordii* (tung oil tree) is a promising industrial crop. Unfortunately, the devastating *Fusarium* wilt disease has caused its great losses, while its sister species (*Vernicia montana*) is remarkably resistant to this pathogen. However, the genetic mechanisms underlying this difference remain largely unknown. We here generated comparative transcriptomic atlases for different stages of *Fusarium oxysporum* infected *Vernicia* root. The transcriptomes of *V. fordii* and *V. montana* were assembled *de novo* and contained 258,430 and 245,240 non-redundant transcripts with N50 values of 1776 and 2452, respectively. A total of 44,310 pairs of putative one-to-one orthologous genes were identified in *Vernicia* species. Overall, the vast majority of orthologous genes shared a remarkably similar expression mode. The expression patterns of a small set of genes were further validated by quantitative real-time PCR. Moreover, 157 unigenes whose expression significantly correlated between the two species were defined, and gene set enrichment analysis indicated roles in increased defense response and in jasmonic and salicylic acid signaling responses during pathogen attack. Co-expression network analysis further identified the 17 hub unigenes, such as the serine/threonine protein kinase D6PK, leucine-rich repeat receptor-like kinase (LRR-RLK), and EREBP transcription factor, which play essential roles in plant pathogen resistance. Intriguingly, the expression of most hub genes differed significantly between *V. montana* and *V. fordii*. Based on our results, we propose a model to describe the major molecular reactions that underlie the defense responses of resistant *V. montana* to *F. oxysporum*. These data represent a crucial step toward breeding more pathogen-resistant *V. fordii*.

## Introduction

Renewable biofuel has recently garnered much interest as a result of increased environmental awareness and the impact of fossil fuel-based energy. *Vernicia fordii* (Hemsl.) Airy-Shaw (*Aleurites fordii* Hemsl.) and *Vernicia montana* Lour. (*A. Montana* (Lour.) Wils.) belong to Euphobiaceae; this family includes many biomass-accumulating species, such as the physic nut (*Jatropha curcas* L.), castor bean (*Ricinus communis* L.), cassava (*Manihot esculenta* Crantz) and rubber tree (*Hevea brasiliensis*). *Vernicia fordii* and *Vernicia montana* are two Chinese native tung oil trees, and the oil that is refined from the seeds of *Vernicia* species has for centuries been used for fuel and industrial purposes. It was used as a fuel in ancient times and now is widely used in the production of paints, high-quality printing, plasticizers, and synthetic rubber. It finds particular use in special paints for the surfaces of weapons, steels, warships and submarines. Moreover, the oil from *Vernicia* species is an important raw material for biodiesel production (Brown and Keeler, 2005; Park et al., 2008; Chen et al., 2010; Shang et al., 2010). China collectively produces approximately 80,000 tons of tung oil per year, or approximately 70–80% of the world market (Zhan et al., 2012). However, this output of tung oil will not meet the needs of the international market in future years.

*Vernicia fordii* (tung oil tree or tung tree) and *V. montana* (wood oil tree) are the two main cultivars. *Vernicia fordii* outperforms *V. montana* in some agronomic traits, as it displays superior oil characteristics and faster maturation, while *V. montana* produces similar oil components and is resistant to wilt disease. Tung wilt disease, caused by the fungal pathogen *Fusarium oxysporum* Schlecht., is considered the most lethal disease of *V. fordii* and is a soil-borne fusarium disease. During infection, plants exhibit leaf chlorosis and slight vein clearing on outer leaflets, which is followed by leaf yellowing and abscission, discoloration of stem vascular tissue and death. To date, the disease cannot be managed unless the tree is grafted with *V. montana* (using *V. montana* as the parental stock), as the latter exhibits high resistance to *F. oxysporum*. They share similarity in morphology, anatomy and karyotype (chromosome number 2n=22=22m). *Fusarium* species are among the most important phytopathogenic and toxigenic fungi. Comparative genomics has revealed that the lineage-specific (LS) genomic region of *F. oxysporum* is related to pathogenicity and that LS transfer can alter strain pathogenicity (Ma et al., 2010). So far, more than 100 plant species have been attacked by *F. oxysporum* (Michielse and Rep, 2009). Recent studies on the molecular basis of *F. oxysporum* pathogenicity have been conducted with a limited number of hosts. The expression of the rice thaumatin-like protein gene in transgenic bananas enhances resistance to *Fusarium* wilt (Mahdavi et al., 2012). The tomato I-3 gene was also reported to confer resistance to *Fusarium* wilt disease (Catanzariti et al., 2015). Similarly, the transcription factor ERF72 conferred resistance to *F. oxysporum* (Chen et al., 2014b). However, little information is available on the genetic mechanism underlying the different resistance profiles of the two closely related *Vernicia* species.

In this study, we took advantage of the phylogenetic proximity between *V. fordii* and *V. montana* to compare the dynamic changes in gene expression during the process of *F. oxysporum* infection. We generated a comparative transcriptome between *V. fordii* and *V. montana* that consisted of four different *F. oxysporum* infection stages for each species.

## Materials and methods

### Pathogen isolation and identification

The pathogen was consistently isolated from the discolored vascular stem tissue of tung trees with wilt disease in Tianlin County, Guangxi Zhuang Autonomous Region, China, and it was then incubated on potato dextrose agar (PDA). Morphological features were first used to identify *F. oxysporum*. The fungus was further identified by PCR-based analysis of the ITS (internal transcribed spacer) and EF-1a (EF-1α gene, translation elongation factor alpha) genes. Specific oligonucleotide primers were used for *Fusarium* identification (Supplemental Table S1) The PCR products were sequenced by Sangon Biotech Company, Shanghai.

Pathogenicity tests were conducted twice in a screen house. Four-week-old plants with wounded roots were submerged for 30 min in a conidial suspension (1 × 10^6^ spores per mL diluted with sterile distilled water), while control plants were dipped in sterile distilled water. Symptoms observed on inoculated seedlings included leaf wilt (5 dpi, days post-inoculation), chlorosis, necrosis and plant death (18 dpi). The pathogen was re-isolated from the stems of affected inoculated plants to confirm the cause of disease. The control plants did not exhibit symptoms. The fungus was identified by morphological features in combination with molecular identification (PCR of the *ITS* and *EF-1a* genes using the primers described above).

### Plant growth and pathogen inoculation

*Vernicia fordii* and *Vernicia montana* plantlets with two or three leaves and approximately 30 cm in height were used. All plantlets were kept under growth-chamber conditions at 26°C with a 16-h light/8-h dark photoperiod. Before inoculation, each plantlet was sterilized in solutions containing 75% ethanol for 1 min, 0.5% sodium hypochlorite for 3 min, and then 90% ethanol for 30 s, followed by three rinses in sterile water.

A spore concentration of 1 × 10^6^ spores per mL, diluted with sterile distilled water, was used to inoculate the plants. Before inoculation, the plants were carefully removed from the soil, and the roots were rinsed with sterile water before being placed into the fungal inoculum for 30 min. After inoculation, the plants grew under growth-chamber conditions at 26°C, with relative humidity over 85% and a 16-h light/8-h dark photoperiod. The plants were then sampled at 2–15 dpi. The root, peel and leaves were cut and immediately frozen in liquid nitrogen and stored at −80°C until retrieval for RNA preparation.

The three time points for harvesting the samples were selected as 2, 8, and 13 dpi according to the symptoms of the seedlings after infection.

### RNA isolation, preparation of cDNA libraries and illumina sequencing

Total RNA was extracted from the plant roots and from the root-stem transition region using the EASYspin plant RNA mini kit (Aidlab, Beijing, China). RNA integrity was confirmed using a UV-Vis Spectrophotometer Q5000 (San Jose, California, USA). All RNA samples displayed a 260/280 ratio of greater than 2.0 and RNA integrity numbers (RIN) ≥8.0. The mRNA was purified from 30 μg of total RNA using Sera-mag Magnetic Oligo (dT) Beads (Illumina, Inc., San Diego, CA, USA) and was then fragmented into small pieces using divalent cations at an elevated temperature. The cDNA library was sequenced on the Illumina HiSeq™ 2000 platform, and both ends of the inserts were sequenced. The 125-bp raw PE reads were generated by the Illumina Genome Analyzer II system (Illumina, Inc., San Diego, CA, USA).

For the RNA-Seq experiments, 4-week-old seedlings of resistant *V. montana* (M) and susceptible *V. fordii* (F) were each treated with *F. oxysporum*. According to the symptoms of the seedlings infected with the pathogen, 4 stages were defined for each species. The stages included untreated *V. fordii* (F0) and *V. fordii* infected with *F. oxysporum* at an early stage (F1), in the middle stage (F2) and at a late stage (F3); they also included untreated *V. montana* (M0) and *V. montana* infected with *F. oxysporum* at an early stage (M1), in the middle stage (M2) and at a late stage (M3). Three biological replicates were taken for each treatment. Therefore, in total, 24 RNA libraries were constructed and sequenced. RNA samples from the root and root-stem transition region were prepared for RNA-seq using Illumina HiSeq™ 2000.

Equal amounts of RNA samples were then pooled for cDNA synthesis and RNA-seq. Before library construction, ribosomal RNA was removed; the cDNA libraries were constructed and sequenced by LC-Bio Co., Ltd, Hangzhou, China. Briefly, 10 μg of total RNA for each group was used for library construction using the Truseq™ RNA sample prep kit (Illumina, USA). The constructed DNA template was then enriched by PCR amplification (15 cycles). Amplicons were collected and purified by electrophoresis on gels made from Certified Low Range Ultra Agarose (Bio-Rad, USA). Before sequencing, the cDNA libraries were quantified using a TBS-380 micro fluorometer with the Picogreen reagent (Invitrogen, USA). Clone clusters were generated with an Illumina cBot using the Truseq PE Cluster Kit v3-cBot-HS, and high-throughput sequencing was performed on an Illumina Miseq sequencer using the Truseq SBS Kit v3-HS for 200 cycles.

### *De novo* assembly of the transcriptome

By using Trinity 30, a *de novo* strategy was adopted to assemble the transcriptome of *V. fordii* and *V. montana* based on a total of 64.5 and 70 GB RNA-seq data (eight samples with three biological replicates for each). N50 number, average length, max length, and contig number during different length interval were all been calculated (Supplemental Table S2).

For the RNA-Seq experiments, 4-week-old seedlings of resistant *V. montana* (M) and susceptible *V. fordii* (F) were each treated with *F. oxysporum*. According to the symptoms of the seedlings infected with the pathogen, 4 stages were defined for each species. The stages included untreated *V. fordii* (F0) and *V. fordii* infected with *F. oxysporum* at an early stage (F1), in the middle stage (F2) and at a late stage (F3); they also included untreated *V. montana* (M0) and *V. montana* infected with *F. oxysporum* at an early stage (M1), in the middle stage (M2) and at a late stage (M3). Three biological replicates were taken for each treatment. Therefore, in total, 24 RNA libraries were constructed and sequenced.

Total RNA was extracted from the plant roots and from the root-stem transition region using the EASYspin plant RNA mini kit (Aidlab, Beijing, China) and the QIAGEN RNeasy plant mini kit (QIAGEN, Valencia, CA). RNA integrity was confirmed using a Bioanalyzer 2100 and the RNA 6000 Nano Lab Chip Kit (Agilent, CA, USA). All RNA samples displayed a 260/280 ratio of greater than 2.0 and RNA integrity numbers (RIN) ≥ 7.0. The mRNA was purified from 5 μg of total RNA using oligo-dT-coupled magnetic beads (Invitrogen) and was then fragmented into small pieces using divalent cations at an elevated temperature. The cleaved RNA fragments were reverse-transcribed to create the final cDNA library according to the protocol for the mRNA-seq sample preparation kit (Illumina, San Diego, USA). Finally, we performed PE sequencing on an Illumina Hiseq2000/2500 at LC-Bio Co., Ltd, Hangzhou, China following the vendor's recommended protocol.

### Sequence functional annotation and classification

To determine the functional annotation of the unigenes, a BLASTX search was performed against the NCBI Nr database with an *E*-value ≤ 10^−5^ and other databases, including SwissProt, Protein Information Resource (PIR), Protein Research Foundation (PRF) and Protein Data Bank (PDB). A BLASTN search was performed against the NCBI Nt database, GenBank, RefSeq, and PDB using a protein query. The CDS (Coding sequences) of unigenes were predicted using GENScan software. The ORFs were identified as the nucleotide sequence or as the protein translation provided by the “GetORF” program from the EMBOSS software package (Rice et al., 2000). The Blast2GO program was used to assign GO terms with an *E*-value ≤ 10^−5^ including molecular functions, biological processes, and cellular components (Conesa et al., 2005; Conesa and Götz, 2008). KEGG pathways were retrieved from KEGG web server (http://www.genome.jp/kegg/). The output of the KEGG analysis includes KO assignments and KEGG pathways that are populated with the KO assignments (Kanehisa et al., 2004).

### Identification of orthologous genes between *V. fordii* and *V. montana*

A reciprocal-best-BLAST-hits (RBH) method with relatively strict filters was applied to identify orthologous genes between *V. fordii* and *V. montana* based on the large-scale transcriptome sequencing. To identify and remove possible paralogous genes, all sequence pairs were first BLAST searched in GenBank by blastp, and several pairs of sequences mapped to the same protein. Next, each sequence from *V. montana* was initially searched against all sequences from *V. fordii* using Blastn, and conversely each sequence from *V. fordii* was searched against all sequences of *V. montana*. Pairs of sequences that were longer than 300 bp and were the best hits for each other in the two species were regarded as putative orthologous genes. Finally, a total of 44,310 pairs of sequences were selected as one-to-one orthologous genes in *V. fordii* and *V. montana*.

### Estimation of synonymous and non-synonymous substitution rates between orthologs

To calculate the synonymous (Ks) and non-synonymous (Ka) substitution rate for each orthologous gene pair in *V. fordii* and *V. montana*, an e-value significance threshold of 10^−5^ was applied, and each member of a pair of sequences was first searched against all plant protein sequences available in GenBank using Blastx; the CDSs of each orthologous gene pair were determined based on the best alignment regions. After removing short (<300 bp) and unexpected stop codon(s)-containing CDSs, the rates of synonymous and non-synonymous substitutions were estimated using the maximum likelihood method implemented in the codeml program under the F3 × 4 model (Zhang et al., 2006; Langfelder and Horvath, 2008).

### Gene expression validation by strand-specific qPCR

To validate the RNA-seq results in terms of gene expression, qPCR was performed using a 7500 Real-Time PCR System (Applied Biosystems, CA, USA) and a SYBR® Premix Ex Taq™ Kit (TaKaRa, Tokyo, Japan). One microgram of RNA was reverse-transcribed using SuperScript III Reverse Transcriptase (Invitrogen) for cDNA synthesis. Primers specific for *Actin7a* (Han et al., 2012) were used to standardize the cDNA. The gene-specific primers (Supplemental Table S1) were used to amplify the corresponding genes. PCR reactions were prepared in 20 μL volumes containing 2 μL of 30-fold diluted synthesized cDNA, 10 μL of 2 × SYBR® Premix Ex Taq™, 0.4 μL of 10 μM forward primer, 0.4 μL of 10 μM reverse primer, 0.4 μL of 50 × ROX reference dye and 6.8 μL of sterile distilled water. The cycling conditions were set as recommended by the manufacturer (30 s at 95°C and then 40 cycles of 95°C for 5 s and 60°C for 34 s). The specificity of amplification was verified by melting curve analysis (60–95°C) after 40 PCR cycles. The experiments included three biological replicates for each treatment and four technical replicates for each biological replicate and the final threshold cycle (Ct) values were set as the mean values according to Pfaffl (2001).

### Data mining

To identify gene interactions, we applied a systems biology approach using an R package for weighted gene co-expression network analysis (WGCNA), which converts co-expression measures into connection weights. We selected the genes in two steps: first, we selected genes that showed significant correlation with a pathogen infection stage (false discovery rate, FDR < 0.01, the *p*-value of Pearson's correlation efficiency was corrected by the BH method); from the remaining genes, we then selected the top 2000 most variable genes based on their coefficients of variance. We input the expression profiles of these selected genes to construct weighted gene co-expression modules using the WGCNA R package (Langfelder and Horvath, 2008). For the gene expression correlations in all of the samples of *V. fordii* and *V. montana*, the log_2_ FPKM (the expected number of fragments per kilobase of transcript sequence per millions base pairs sequenced) values for pairs of orthologous genes were calculated.

## Results

### Sister *Vernicia* species respond differently to *F. oxysporum*

**Figure 1:**
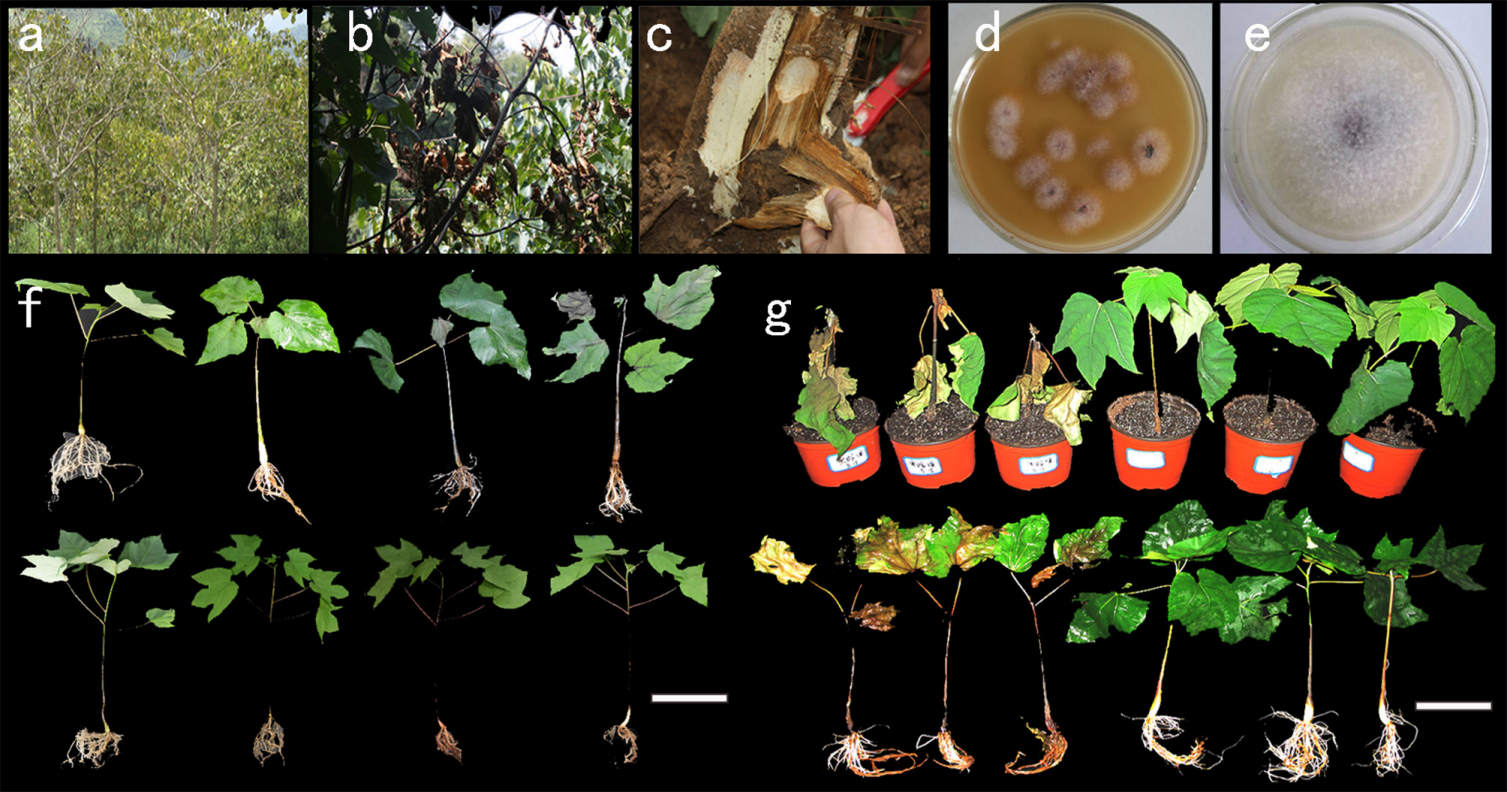
**Tung wilt pathogen isolation and pathogenicity testing in ***V. fordii*** and ***V. montana***. (A)** A tung forest attacked by wilt disease; **(B)** a *V. fordii* tree with *Fusarium* disease; **(C)** a longitudinal section of a *V. fordii* tree with wilt disease; **(D)** isolation of pathogens from the wilt-diseased tung tree; **(E)** a further pure culture of the potential fungal pathogen; **(F)** seedlings of *V. fordii* and *V. montana* infected with the tung wilt pathogen *F. oxysporum* at different stages: the upper row shows *V. fordii* seedlings, and the lower row shows *V. montana* seedlings; **(G)** seedlings of *V. fordii* and *V. montana* severely infected with *F. oxysporum*. Scale bar = 10 cm.

*Vernicia fordii* and *Vernicia montana* responded differently to the pathogen *F. oxysporum* Schlecht. To characterize differences in the pathogen infection processes between *V. fordii* and *V. montana*, 4-week-old seedlings of resistant *V. montana* (M) and susceptible *V. fordii* (F) were each treated with *F. oxysporum*. According to the symptoms of the pathogen-infected seedlings, four stages were defined for each species. The stages included untreated *V. montana* (M0) and *V. montana* infected with *F. oxysporum* in the early stage (M1), in the middle stage (M2) and in the late stage (M3); they also included untreated susceptible *V. fordii* (F0) and *V. fordii* infected with *F. oxysporum* in the early stage (F1), in the middle stage (F2) and in the late stage (F3). An overview of seedlings with different treatments is shown as Figure 1.

### Output statistics of the transcriptomes of *V. fordii* and *V. montana* inoculated with *F. oxysporum*

Using Illumina HiSeq™ 2000 sequencing, approximately 516,422,088 and 559,181,176 clean paired-end (PE) reads were generated from 24 libraries of *V. fordii* (F0, F1, F2, and F3) and *V. montana* (M0, M1, M2, and M3), respectively. The number of clean reads ranged from 29.2 to 66.5 million across libraries F0, F1, F2, and F3, which were assembled together for *V. fordii*, and the read number ranged from 30.6 to 66.9 million across libraries M0, M1, M2, and M3, which were assembled for *V. montana* (Table 1). The high-quality reads are available in the NCBI Gene Expression Ominibus (GEO) with accession number GSE80228. *De novo* assembly of *V. fordii* yielded a total of 258,435 transcripts with a mean length of 1010 bp, which integrated into 133,304 unigenes with an average length of 648 bp. For *V. montana, de novo* assembly yielded a total of 245,240 transcripts with a mean length of 1376 bp, which integrated into 111,278 unigenes with an average length of 726 bp. The N50 sizes of the unigenes were 1033 and 1289 in *V. fordii* and *V. montana*, respectively (Table 2). The overall GC content of *V. fordii* in coding regions was 40.60%, consistent with that of *V. montana* (39.60%). The output statistics for sequencing and the mapping statistics for each sample stage of *V. fordii* and *V. montana* infected with *F. oxysporum* are shown in Supplemental Table S2.

**Table 1:** **Output statistics of sequencing and mapping stats for each stage samples in ***V. fordii*** and ***V. montana*** infencted with ***F. oxysporum*****.

	**Valid data reads**	**Q20%**	**Q30%**	**GC%**		**Valid data reads**	**Q20%**	**Q30%**	**GC%**
F0	47,271,474	98.59	95.80	91.94	M0	42,329,318	98.65	95.63	91.48
F0	66,515,356	99.25	100	100	M0	66,859,072	99.25	100.00	100.00
F0	29,272,386	98.49	94.95	90.39	M0	41,093,632	98.65	95.08	90.63
F1	31,347,672	98.87	95.48	91.22	M1	39,967,224	97.43	96.37	93.10
F1	63,432,696	99.20	100	100	M1	65,782,492	99.28	100.00	100.00
F1	29,620,658	98.82	95.39	91.10	M1	39,077,922	98.91	95.35	91.08
F2	29,482,698	97.66	95.88	92.04	M2	30,558,196	99.00	94.76	89.87
F2	64,285,262	99.21	100	100	M2	66,242,736	99.33	100.00	100.00
F2	28,992,470	98.57	94.79	90.03	M2	32,451,888	98.55	95.97	92.26
F3	29,419,992	99.26	95.06	90.48	M3	36,548,912	97.97	95.96	92.27
F3	64,236,976	99.43	100	100	M3	64,240,712	99.28	100.00	100.00

*Q20: percentage is the proportion of nucleotides with a quality value >20 in reads*.

**Table 2:** **Assembly statistics (all the clean reads from the 4 cDNA libraries of each species were together assembled)**.

		**All**	**Median GC%**	**Mean GC%**	**Min length**	**Median length**	**Mean length**	**Max length**	**Total assembled bases**	**N50**
F	Unigene	133,304	40.60	41.62	201	351	648	23,838	86,436,155	1033
	Transcript	258,435	40.60	41.62	201	558	1010	23,838	261,063,231	1776
M	Unigene	111,278	39.60	39.85	201	368	726	23,849	80,872,199	1289
	Transcript	245,240	39.60	39.85	201	801	1376	23,849	337,505,963	2452

*N50: unigene length-weighted median*.

The BLAST top-hit species distribution showed that the assembled transcript annotation in *V. fordii* had the highest homology to *J. curcas* (34.1%), *R. communis* (14.6%), *Populus trichocarpa* (5.9%), *Theobroma cacao* (2.6%), *Vitis vinifera* (2.5%), *Citrus sinensis* (1.7%) and others (38.6%) (Supplemental Figure S1A). The assembled transcript annotation in *V. montana* displayed the highest homology to *R. communis* (36.2%), *Vitis vinifera* (8.1%), *P. trichocarpa* (7.9%), *T. cacao* (3.8%), *C. sinensis* (1.8%) and others (42.2%) (Supplemental Figure S1B).

For the purpose of functional annotation and classification, the coding sequences (CDS) of 67,961 (50.98%) and 51,924 (46.66%) unigenes were successfully predicted in the *V. fordii* and *V. montana* transcriptomes, respectively. A total of 133,304 and 111,278 unigenes in *V. fordii* and *V. montana*, respectively, were annotated from different databases. By gene ontology (GO) analysis, 46,210 (34.67%) unigenes were assigned into 17 classes in *V. fordii*, while 36,507 (32.81%) unigenes were classified into 19 classes in *V. montana*. By searching against the Kyoto Encyclopedia of Genes and Genomes Pathway database (KEGG), the unigenes were assigned to 279 KEGG pathways in both *V. fordii* and *V. montana* (Table 3).

**Table 3:** **Transcripts and unigenes annotation statistics for the transcriptom of ***V. fordii*** (F) and ***V. montana*** (M)**.

		**Total**	**Swiss-prot**	**Nr**	**Pfam**	**KEGG**	**KOG**	**GO**
F	Transcripts	174,545 (67.54%)	117,572 (45.49%)	163,870 (63.41%)	143,026 (55.34%)	87,874 (34.00%)	146,484 (56.68%)	105,083 (40.66%)
	Unigenes	76,924 (57.71%)	50,220 (37.67%)	63,225 (47.43%)	60,310 (45.24%)	39,367 (29.53%)	44,074 (33.06)	46,210 (34.67)
M	Transcripts	175,571 (71.59%)	108,050 (44.06%)	172,292 (70.26%)	140,300 (57.21%)	165,367 (67.43%)	143,551 (58.53%)	95,803 (39.06%)
	Unigenes	60,842 (54.68%)	40,013 (35.96%)	53,395 (47.98%)	47,593 (42.77%)	29,847 (26.82%)	34,528 (31.03%)	36,507 (32.81%)

### *Vernicia* transcriptomes display high quality and are comparable between *V. fordii* and *V. montana*

To evaluate the consistency among biological replicates, the expected number of fragments per kilobase of transcript sequence per millions base pairs sequenced (FPKM) expression data were tested by correlation analysis, and all of the correlation coefficients between biological replicates were calculated. There was a strong correlation for triplicate samples, which reached average Pearson correlations *R*^2^ of 0.835 and 0.870 in *V. fordii* and *V. montana*, respectively (Supplemental Figure S2). Biological replicates at each sample stage clustered closely and were highly correlated.

To evaluate the conservation of gene expression patterns between *V. fordii* and *V. montana*, a reciprocal-best-BLAST-hits (RBH) method (Ward and Moreno-Hagelsieb, 2014) was applied, and the similarity threshold was set to 90%. Using this method, we defined 44,310 pairs of one-to-one orthologous unigenes between the *V. fordii* (57.60%, 44,310/76,924) and *V. montana* (72.83%, 44,310/60,842) transcriptomes.

### Global changes in gene expression in resistant *V. montana* and susceptible *V. fordii*

**Figure 2:**
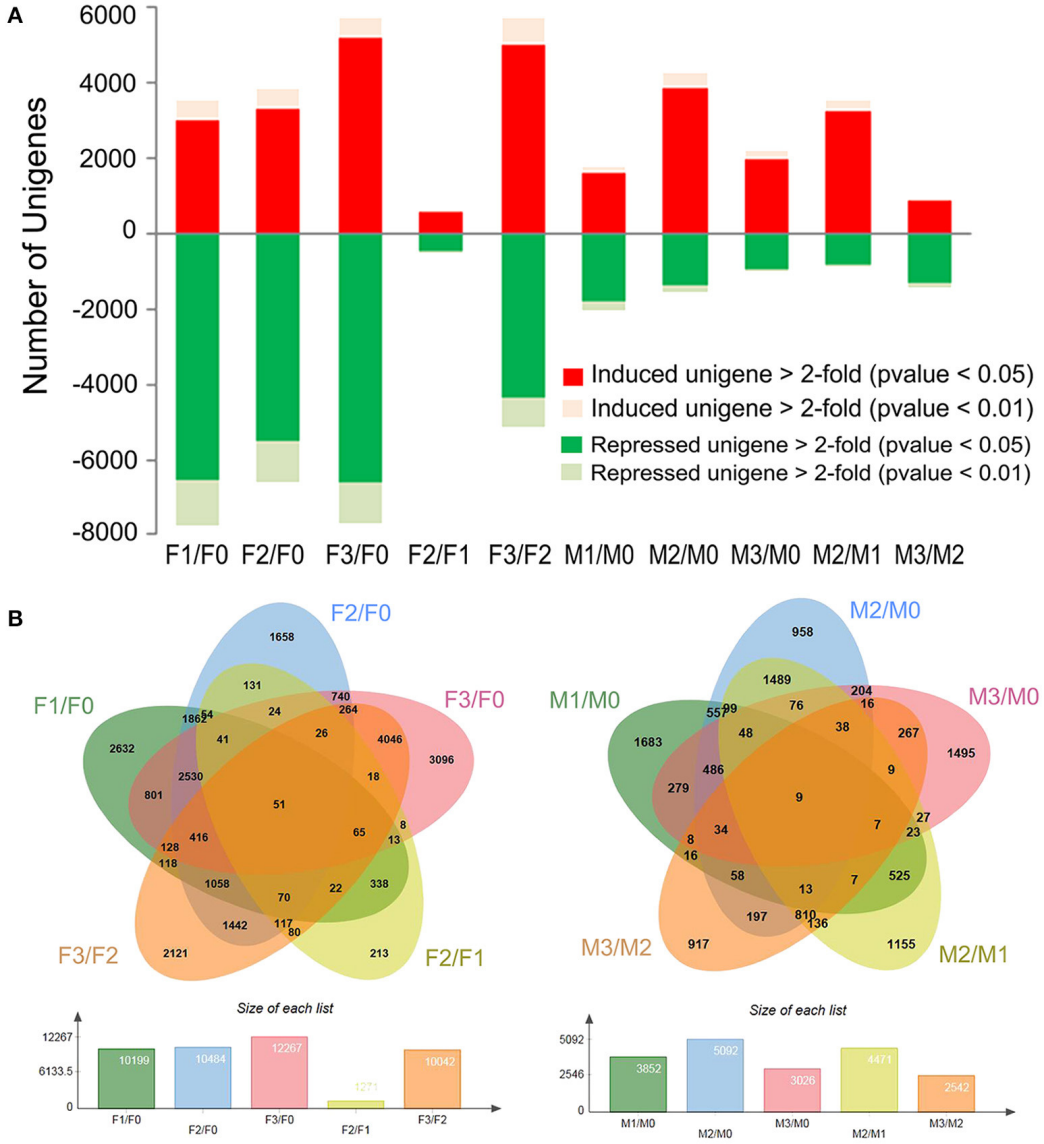
**Global changes in gene expression in resistant ***V. montana*** and susceptible ***V. fordii*** during ***F. oxysporum*** infection. (A)** The number of >2-fold induced and repressed unigenes (*p* < 0.05 and 0.01) for F1 vs. F0, F2 vs. F1 and F3 vs. F2 and for M1 vs. M0, M2 vs. M1 and M3 vs. M2; **(B)** the numbers of commonly and specifically induced and repressed unigenes at different stages of *F. oxysporum* infection in the two species are shown in the overlapping and non-overlapping regions, respectively.

To identify the unigenes with differential gene expression (DGE) during stages F0-F3 and M0-M3, a significance threshold of 0.05 was applied using Edge R package (Robinson et al., 2010). The differential expression modes between the two species were thus revealed (Figure 2). For V. fordii, 3002 and 6548 unigenes were upregulated and repressed, respectively, at the early stage (F1 vs. F0) after infection. At the next stage, 557 and 456 unigenes were significantly induced and repressed, respectively (F2 vs. F1). However, for *V. montana*, 1640 and 1786 unigenes were significantly induced and repressed, respectively, in the early stage (M1 vs. M0), while 3218 and 813 unigenes were significantly induced and repressed, respectively, at the subsequent stage (M2 vs. M1) (Figure 2, Supplemental Figure S3). It seemed that many genes responded in the early stage F1 in *V. fordii*, while a large proportion of genes was induced in the later M2 stage in *V. montana*.

**Figure 3:**
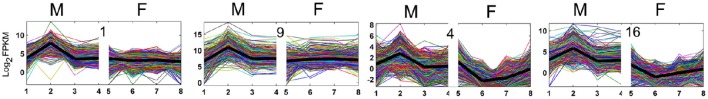
**Clustering of significantly differential expressed orthologous genesusing ***k-means*** clustering method according to the gene expression profiles in ***V. montana*** (M) and ***V. fordii*** (F) infected with the pathogen ***F. oxysporum*****. The differential expression modes were classified into twenty clusters in *V. montana*, and the corresponding orthologous genes in *V. fordii* were analyzed for their expression modes (Supplemental Figure S4).

The one-to-one orthologous gene analysis facilitated a comparison of gene expression in the two species. To investigate global changes in expression patterns, we performed *k-*means clustering of the gene expression profiles for orthologous genes in susceptible *V. fordii* and resistant *V. montana* during pathogen infection. We classified the different expression modes into 20 clusters that showed distinctive expression patterns in *V. montana* and included the corresponding orthologous genes in *V. fordii* (Figure 3, Supplemental Figure S4, and Supplemental Table S3). Our comparative transcriptome analysis revealed that a high proportion of orthologous genes exhibited similar expression patterns (Supplemental Figure S4). Only a fraction of orthologous genes, including clusters 4, 12, and 16, showed divergent expression patterns. Notably, in cluster 4 and 16, the genes showed a sharp increase in *V. montana* while exhibiting a clear decrease in *V. fordii*, thereby indicating different roles for these genes in the two species when responding to the pathogen. In cluster 1 and 9, genes exhibited dramatically increased (log_2_ FPKM ≥ 5) in *V. montana*, while expressed in an invariant way in *V. fordii* (Figure 3).

**Figure 4:**
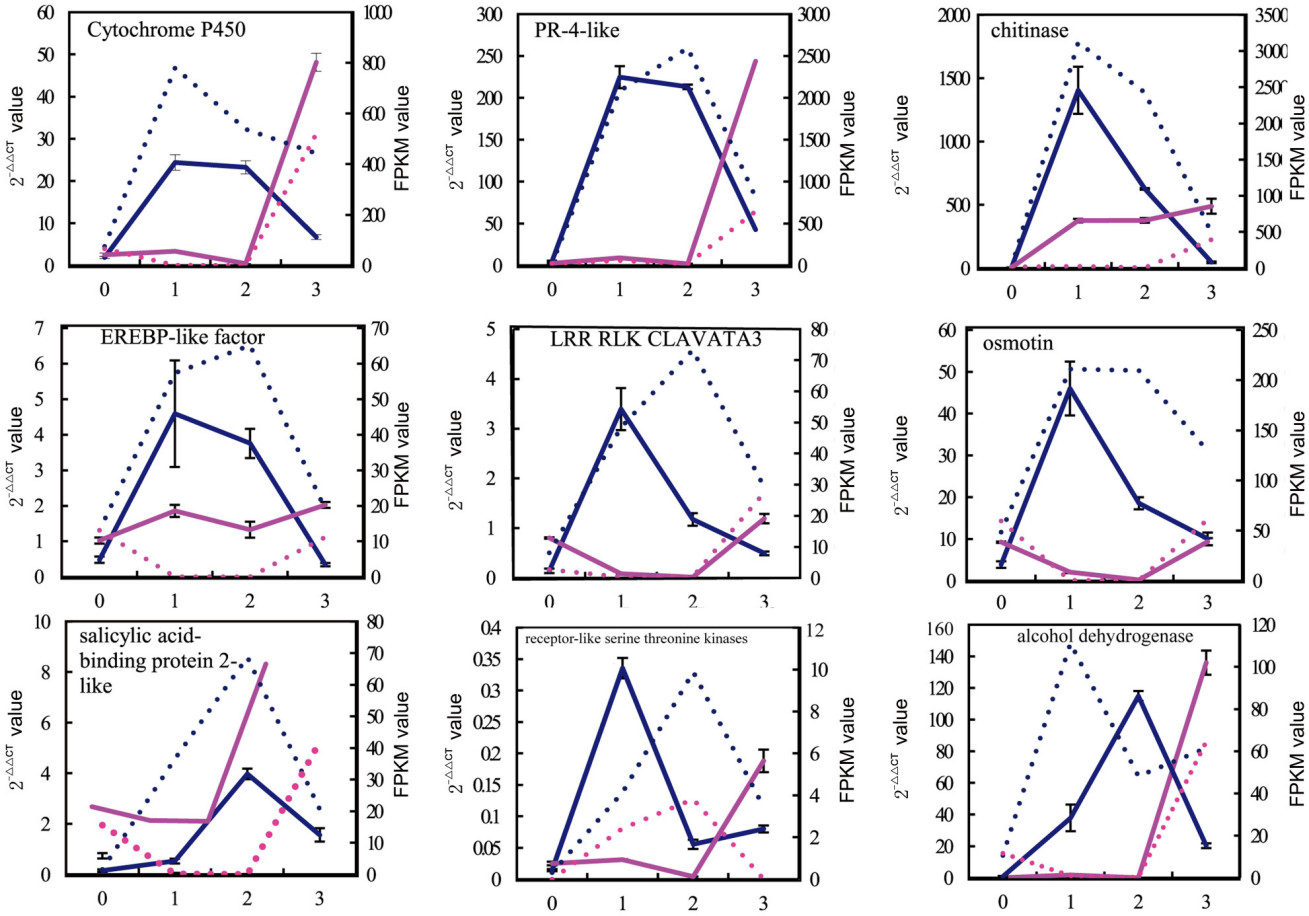
**Verification of the expression profiles of 9 pairs of unigenes by qPCR**. In total, 30 candidate genes were chosen to validate using qPCR from among the different k-means clusters in *V. fordii* and *V. montana* (Supplemental Figure S4). The results of qPCR were generally in accordance with the gene expression profiles in the transcriptome data. X-axis represented the different stage of *F. oxysporum* infection. 0, the stage before infection; 1, the early stage of infection with *F. oxysporum*; 2; the middle stage of infection with *F. oxysporum*; 3, the late stage of infection with *F. oxysporum*. The left Y-axis represented the expression level (2^−ΔΔ*CT*^ value) of genes. The right Y-axis represented the expression level of genes according to the FPKM value. The blue solid line represented the expression level (2^−ΔΔ*CT*^) of genes in *V. montana* (M) using qPCR; the blue dotted line represented the expression level of genes in *V. montana* (M) according to the FPKM value; the red real line represented the expression level (2^−ΔΔ*CT*^) of genes in *V. fordii* (F) using qPCR; the red dotted line represented the expression level of genes in *V. fordii* (F) according to the FPKM value.

To validate the gene expression profiles, quantitative RT-PCR analyses were conducted for 30 candidate genes that were chosen from different *k-*means clusters in *V. fordii* and *V. montana* (Figure 4, Supplemental Figure S5). These unigenes encoded proteins such as PR-4-like protein, chitinase, EREBP-like transcription factor, the LRR-RLK CLAVATA3, salicylic acid-binding protein 2-like, receptor-like serine/threonine kinases, cytochrome P450, alcohol dehydrogenase and osmotin, among others. The results of the qPCR analyses were generally in accordance with the gene expression profile shown in the transcriptome (Supplemental Figure S5).

The GO analysis revealed conservation across most transcripts but significant divergence between the species for a subset of RNAs. In *V. fordii*, nucleoid and protein-binding transcription factor gene expression significantly increased in the early response stage. In *V. montana*, receptor activity and protein tag genes showed strong induction in the early stage. Our study generated a substantial number of transcript sequences during the infection responses of resistant and susceptible *Vernicia* species. The results contributed to the identification of pathways and functional genes in *Vernicia* species that respond to pathogen attack, and they will aid in understanding the mechanisms with which *V. fordii* and *V. montana* respond to *F. oxysporum*.

## Discussion

### Conserved and divergent expression patterns between *V. fordii* and *V. montana* when responding to *F. oxysporum*

To reveal genes that were highly conserved or divergent in response to *F. oxysporum* infection, we performed correlation analysis between orthologous genes. In total, we identified 450 unigenes that were significantly connected (either conserved or divergent, with correlation coefficient cutoffs of *r* > 0.65 or *r* < −0.65, *p* < 0.05) between the two species; they are listed in Supplemental Table S4. Among them, 157 unigenes were significantly negatively associated (*r* < −0.65, *p* < 0.05); they included two Myb transcription factors, mitogen-activated protein kinase 3 (MAPK3), a homeobox-leucine zipper protein, a G-box-binding factor, RIO kinase 2, phytochrome-interacting factor 3 (PIF3) and others. Several Mybs reportedly participate in defending against pathogen attack, including AtMyb30, AtBOS1 (AtMyb108), and TaPIMP1 (Vailleau et al., 2002; Zhang et al., 2012; Corso et al., 2015), yet the associated regulatory mechanisms and signaling processes remain largely unknown. The expression of MAPK3 and a homeobox-leucine zipper protein increased in the later stage of the response to cadmium stress (Yang et al., 2015). A homeobox-leucine zipper gene from sunflower and *Arabidopsis* conferred drought and salt tolerance (Shin et al., 2004; Dezar et al., 2005). The G-box is the cognate cis-element for the bZIP, bHLH, and NAC proteins. bHLH is reportedly a critical protein in plant development, senescence, iron metabolism and regulation of pathogen defense responses, and it leads to the activation of the salicylic acid signaling pathway (Zhang et al., 2013; Aparicio and Pallás, 2016). PIF3 activates light-responsive transcriptional network genes in coordination with the circadian clock and plant hormones to modulate plant growth and development. RIF3 is also a candidate target for manipulating the weed stress response in soybeans, and it regulates the plant response to drought and salt stress in maize (Gao et al., 2015; Horvath et al., 2015).

We found that 293 unigenes showed a significant positive association in their expression in the two species using a two-sample *t*-test (with correlation coefficients of *r* > 0.65, *p* < 0.05; Supplemental Table S4); they included EREBP transcription factors, serine/threonine protein kinases, a receptor-like serine/threonine protein kinase, MAPKKKs, a proline-rich receptor-like protein kinase, a cytochrome P450 and others. EREBP was reported to confer biotic and abiotic stress tolerance in rice (Jisha et al., 2015). Serine/threonine protein kinases and receptor-like serine/threonine protein kinases have been associated with the early stage of plant defense.

**Figure 5:**
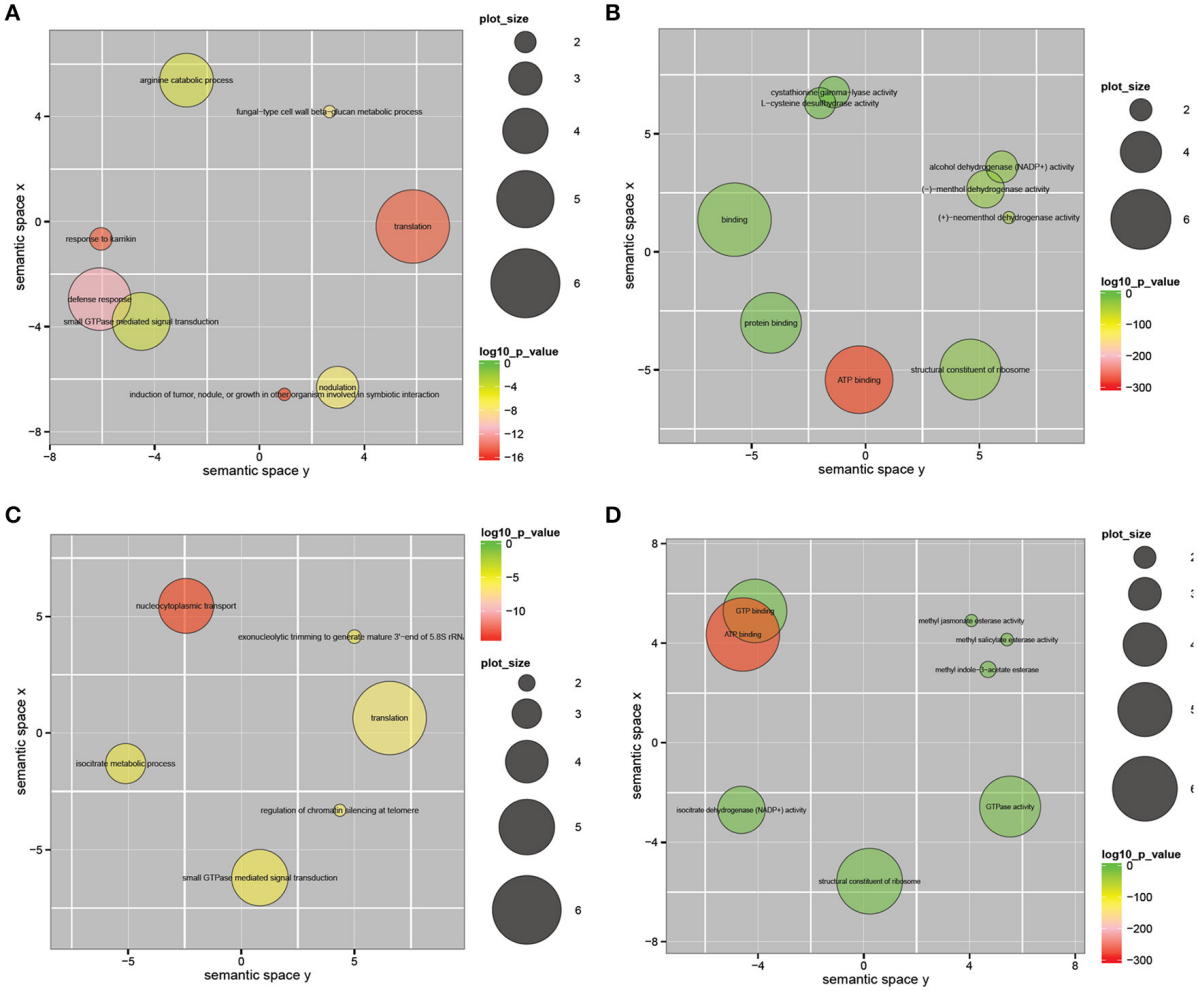
**Gene ontology enrichment analysis of 450 pairs of unigenes that showed significant positive or negative correlation (correlation coefficients ***r*** > 0.65 or ***r*** < −0.65, ***p*** < 0.05) between the gene expression modes in ***V. fordii*** and ***V. montana***. (A)** Significantly positively correlated GO term in biological process; **(B)** significantly positively correlated GO term in molecular function; **(C)** significantly negatively correlated GO term in biological process; **(D)** significantly negatively correlated GO term in molecular function. The scatterplot shows the cluster representatives in a two dimensional space derived by applying multidimensional scaling to a matrix of the GO terms' semantic similarities. Bubble color indicates the log 10 *p*-value (legend in upper right-hand corner); plot size indicates the frequency of the GO term in the underlying GOA database (bubbles of more general terms are larger).

To functionally classify the 450 significantly correlated genes, we applied REVIGO and ggplot2 and observed significant enrichment of these genes in multiple GO categories (Supek et al., 2011). The GO enrichment analysis of the 450 pairs of unigenes with significant positive or negative correlations is shown in Figure 5. The significantly positively correlated GO terms among biological processes included fungal-type cell wall beta-glucan metabolic process, defense response and small GTPase mediated signal transduction, among others (Figure 5A). The significantly positively correlated GO terms among molecular functions included alcohol dehydrogenase (NADP+) activity, (−)-menthol dehydrogenase activity, binding, protein binding, ATP binding and structural constituents of the ribosome (Figure 5B). The significantly negatively correlated GO terms among biological processes included translation, isocitrate metabolic process, regulation of chromatin silencing at telomere and small GTPase mediated signal transduction, among others (Figure 5C). The significantly negatively correlated GO terms for molecular function included methyl jasmonate esterase activity, methyl salicylate esterase activity and isocitrate dehydrogenase (NADP+) activity (Figure 5D).

### Gene co-expression networks and pathways in resistant *V. montana*

**Figure 6:**
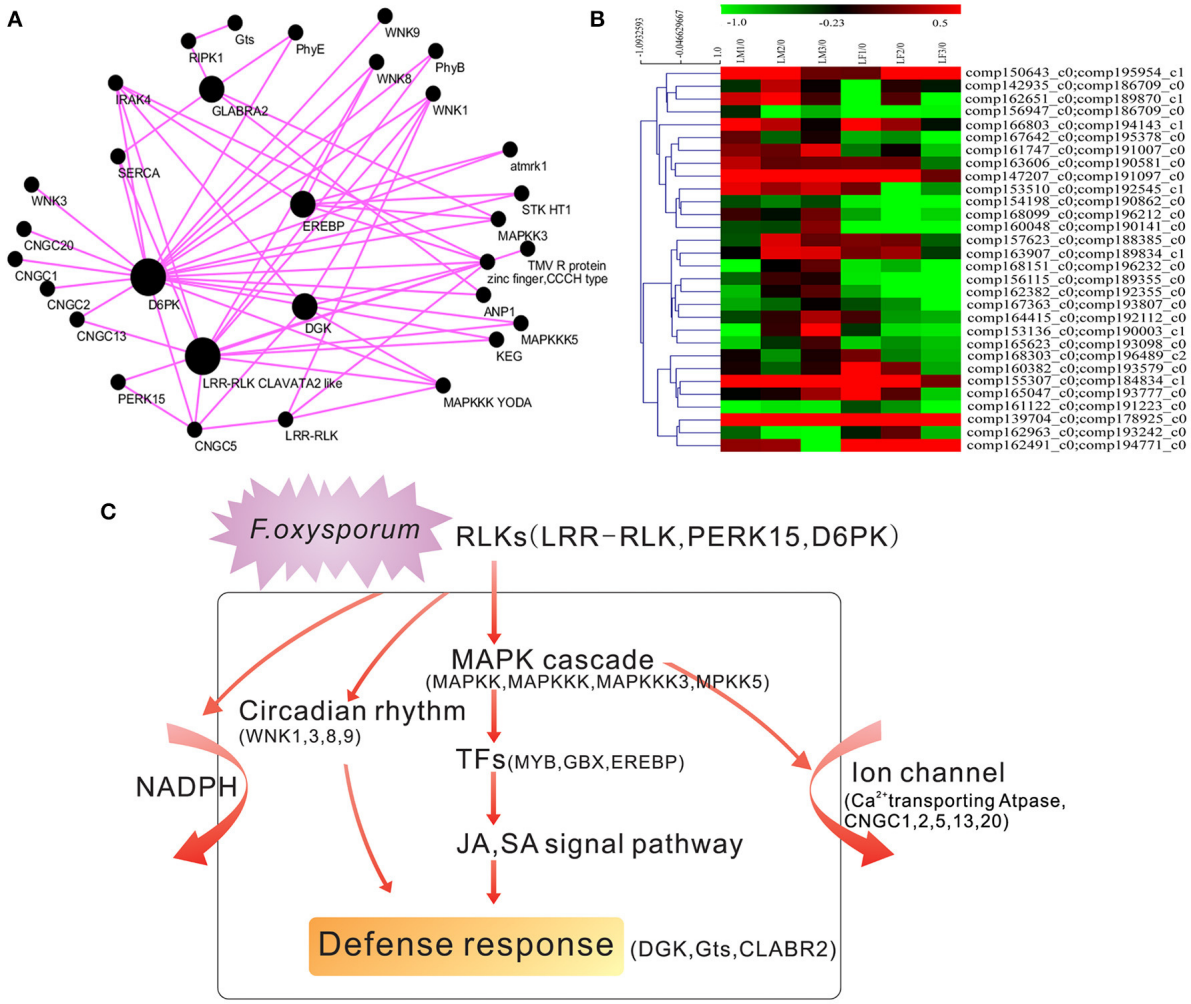
**Hub gene identification and model for the defense response in ***V. montana***. (A)** Identification of resistance-related hub genes in *V. montana* responding to *F. oxysporum*. The connections were drawn using Cytoscape. The genes with a weighted cutoff value >0.50 are shown. Each node represents a gene. Black nodes indicate hub genes. Bigger nodes indicate more connections. **(B)** Expression patterns of pairs of hub genes in the two species during infection with *F. oxysporum*. **(C)** A model to describe the major molecular reactions underlying the defense responses to *F. oxysporum* in resistant *V. montana*.

To give a higher-order interpretation of gene expression relationships and to identify modules of co-expressed genes that were functionally related, we implemented a systems biology approach and analyzed the gene expression profiles of 24 susceptible and resistant transcriptomes. The centralized genes, called hub genes, were highly connected with peripheral genes. We raised the weighted cutoff value to >0.50 to identify hub genes with the strongest connections to other genes. Under this criterion, we visualized the 50 most highly connected genes as hub genes. Among them, 26 hub genes were functionally annotated as hypothetical proteins, and 17 unigenes playing essential roles in plant pathogen resistance (Supplemental Table S5). We further used the software Cytoscape 3.2.1 to build the co-expressed gene network (Figure 6A). As the Figure 6A showed, among the top 50 hub genes, the serine/threonine protein kinase D6PK and the LRR-RLK CLAVATA2 were strongest, with 2611 and 1752 connections, respectively (Figure 6A). LRR-RLKs are reportedly involved in plant defense responses against various classes of pathogens and may initiate the activation of the MAPK pathway, WRKY transcription factors, ion channels and the NADPH oxidase complex (Yang et al., 2012). The LRR-RLK2 CLAVATA has been reported to confer enhanced disease resistance to bacterial wilt (Hanemian et al., 2016). Diacylglycerol kinase (DGK), which confers resistance to *Magnaporthe grisea* (Zhang et al., 2008) was also identified as a hub gene with 1253 connections. Furthermore, glycosyltransferase (Gts), which is reportedly involved in resistance to *F. oxysporum* (Lorenc-Kukuła et al., 2009), was also identified as a hub gene with 185 connections. The homeobox-leucine zipper protein GLABRA2 was recognized as a hub gene with 848 connections, and the EREBP transcription factor was defined as a hub gene with 666 connections.

Among the top 50 hub genes, the expression modes of Gts, the proline-rich receptor-like protein kinase PERK15 and an EREBP-like factor in *V. fordii* and *V. montana* were significantly and positively correlated during the infection (correlation coefficient *r* > 0.65, *p* < 0.05). However, the expression of the homeobox-leucine zipper protein GLABRA2 was significantly negatively correlated between the two species (correlation coefficient *r* < −0.65, *p* < 0.05). Genes with higher connectivity tended to have a stronger correlation with resistance to *F. oxysporum*, suggesting a potentially important role of highly connected genes (hub genes) in the resistance of *V. montana* to *F. oxysporum*.

The expression patterns of the hub genes were analyzed in the two species during infection with *F. oxysporum* according to their FPKM values. The expression of 30 of hub genes showed induction in *V. montana* but repression in *V. fordii* during infection with the pathogen *F. oxysporum* (Figure 6B).

To functionally classify these significant genes, we observed the enrichment of these genes in several KEGG pathways. Significant pathways included the MAPK signaling pathway, plant-pathogen interaction, circadian rhythm, the calcium signaling pathway and apoptosis. Hierarchical clustering revealed major modules that exhibited totally different expression patterns during the infection process. Enrichment for these categories indicated that the KEGG pathways described above play a major role in driving transcriptional variation among *Vernicia* species infected with *F. oxysporum*.

### Evolution analysis reveal conservation and diversification between *Vernicia* orthologs

To define the molecular evolution of orthologous genes shared by *V. fordii* and *V. montana*, particularly genes undergoing purifying or positive selection, we calculated the ratio of synonymous (Ks) and non-synonymous (Ka) substitution rates (Ka/Ks) for each pair of orthologous genes (Yang and Bielawski, 2000). Ka/Ks = 1 signifies neutral evolution, Ka/Ks > 1 is a sign of positive (adaptive) selection, and Ka/Ks < 1 is a sign of negative (purifying) selection. Our results showed that the mean value of Ka/Ks for orthologous gene pairs was 3.32. The majority of sequences (90.17%, 6020/6676) had a Ka/Ks ratio of <1, while a small portion of genes (9.83%, 656/6676) had a Ka/Ks ratio of >1, suggesting that most of the orthologous genes are undergoing purifying selection. We also identified 208 sequences under accelerated evolution, with ratios of Ka/Ks > 1 and *p*-value < 0.05, among which 167 sequences showed ratios of Ka/Ks > 2 and *p*-value < 0.05 (Supplemental Table S6). These fast-evolving transcripts may be useful for identifying genes that perhaps strongly underwent positive selection during the evolutionary process and that may be responsible for speciation in the Camellia lineage. Notably, the proline-rich receptor-like protein kinase PERK15 was not only significantly positively correlated between *V. fordii* and *V. montana* during *F. oxysporum* infection, but it also had a Ka/Ks ratio of >1.

### Hypothesized molecular reactions in resistant *V. montana* responding to *F. oxysporum*

*F. oxysporum*, a hemi-biotrophic root pathogen, infects a number of plants, including cotton, tomato and banana. Some *Arabidopsis* mutants with impaired jasmonic acid (JA) signaling are more susceptible to other pathogens but show strong resistance to *F. oxysporum* (Anderson et al., 2004; Kidd et al., 2009). This result suggests that *F. oxysporum* disguises itself as a beneficial microbe, activating the JA pathway to gain entry into the cell as a hemi-biotrophic pathogen (Chen et al., 2014a).

The LRR-RLK2 CLAVATA was defined as a main hub gene in our study. Thus, hierarchical clustering of the expression modes of all pairs of LRR-RLKs in *V. fordii* and *V. montana* was conducted (Supplemental Figure S6). At the bottom of the hierarchical clustering, the expression of a small set of LRR-RLKs was induced in *V. montana* but repressed in *V. fordii*. LRR-RLKs have been reported to have key roles in pathogen recognition, and they may lead to the activation of ion channels, the NADPH oxidase complex and MAPK cascades after trans-phosphorylation and phosphorylation events (Yang et al., 2012). Similarly, we conducted gene enrichment analysis on 450 unigenes and also clearly identified the above pathways (Figure 5). Moreover, enrichment analysis of the top genes indicated roles for the MAPK signaling pathway, plant-pathogen interaction, circadian rhythm, the calcium signaling pathway and apoptosis during *F. oxysporum* infection of *V. montana*. Based on our results, we propose a model to describe the major molecular reactions that underlie the defense responses of resistant *V. montana* to *F. oxysporum* (Figure 6C). Upon attack by *F. oxysporum*, protein kinases, such as LRR-RLK CLAVATA, PERK15 and D6PK, act as a first line of defense against the pathogen. A series of signal transduction pathways, including the MAPK, JA, and SA signaling pathways, are then initiated. Meanwhile, circadian rhythms, NADPH and ion channels are activated by receptor like kinases (RLKs) in the plasma membrane. Furthermore, transcription factors, such as Myb, GBS, and EREBP, are activated, thereby finally inducing the expression of a series of defense-related genes, including DGX, Gts, CLABR2, and others. There appears to be some crosstalk in the signaling pathways.

## Accession numbers

The datasets supporting the conclusions of this article are available in the NCBI GEO repository under accession number GSE80228. http://www.ncbi.nlm.nih.gov/gds/

## Author contributions

YC conceived the original screening and research plans; YW supervised the experiments; YC performed most of the experiments; MG, HZ, and QZ provided technical assistance to YC; YC, HY designed the experiments and analyzed the data; YC conceived the project and wrote the article with contributions of all the authors; HY, YW supervised and complemented the writing.

### Conflict of interest statement

The authors declare that the research was conducted in the absence of any commercial or financial relationships that could be construed as a potential conflict of interest.

## Supplementary material

Supplemental Figure S1 **The top BLAST species hits with homology to *V. fordii* (A)** and *V. morntana* **(B)** according to the transcript annotation.

Supplemental Figure S2 **Pearson's correlation heat map of the unigenes (FPKM) of all the samples (n = 3) in *V. fordii* and *V. montana*. F, *V. fordii*; M, *V. Montana***.

Supplemental Figure S3 **A volcano plot using a fold change difference for differential expressed genes in *V. fordii* and *V. montana***.

Supplemental Figure S4 **A high proportion of orthologous genes exhibited similar expression patterns using *k-means* clustering of one-to-one orthologous unigene pairs according to the gene expression profiles in *V. montana* (M) and *V. fordii* (F) infected with the pathogen *F. oxysporum***. The differential expression modes were classified into 20 clustersin *V. montana*, and the corresponding orthologous genes in *V. fordii* were analyzed for their expression modes (the other four clusters expressed significantly different were shown in Figure 3).

Supplemental Figure S5 **Verification of the expression profiles of 21 pairs of unigenes by real-time quantitative RT-PCR**. X-axis represented the different stage of *F. oxysporum* infection. 0, the stage before infection; 1, the early stage of infection with *F. oxysporum*; 2; the middle stage of infection with *F. oxysporum*; 3, the late stage of infection with *F. oxysporum*. The left Y-axis represented the expression level (2^−ΔΔ*CT*^ value) of genes. The right Y-axis represented the expression level of genes according to the FPKM value. The blue solid line represented the expression level (2^−ΔΔ*CT*^) of genes in *V. montana* (M) using qPCR; the blue dotted line represented the expression level of genes in *V. montana* (M) according to the FPKM value; the red real line represented the expression level (2^−ΔΔ*CT*^) of genes in *V. fordii* (F) using qPCR; the red dotted line represented the expression level of genes in *V. fordii* (F) according to the FPKM value.

Supplemental Figure S6 **Comparative expression patterns of pairs of LRR-RLK genes between susceptible *V. fordii* and resistant *V. montana* underlying the response to attack by *F. oxysporum***.

Supplemental Table S1 **Primer sequences of genes amplification and qPCR in the study**.

Supplemental Table S2 **Output statistics of sequencing and mapping stats for each sample stage in *V. fordii* and *V. montana* infected with *F. oxysporum***.

Supplemental Table S3 **Statistical analysis on the k mean clusters of orthologous genes in *V. fordii* and *V. montana***.

Supplemental Table S4 **The list of genes with significantly correlated expression between *V. fordii* and *V. montana* using a two-sample *t*-test (correlation coefficient *r* > 0.65 or *r* < −0.65, *p* < 0.05)**.

Supplemental Table S5 **Identification of the top 50 hub genes with a raised weighted cutoff value >0.50**.

Supplemental Table S6 **The molecular evolution of orthologous genes between *V. fordii* and *V. montana* under accelerated evolution with ratios of Ka/Ks>1 and *p* < 0.05**.
