# Supplementary figures and images for: Comparative Transcriptomics Atlases Reveals Different Gene Expression Pattern Related to Fusarium Wilt Disease Resistance and Susceptibility in Two Vernicia Species
Source: Front Plant Sci. 2016 Dec 27;7:1974. doi: 10.3389/fpls.2016.01974 (PMC5186792; doi:10.3389/fpls.2016.01974)

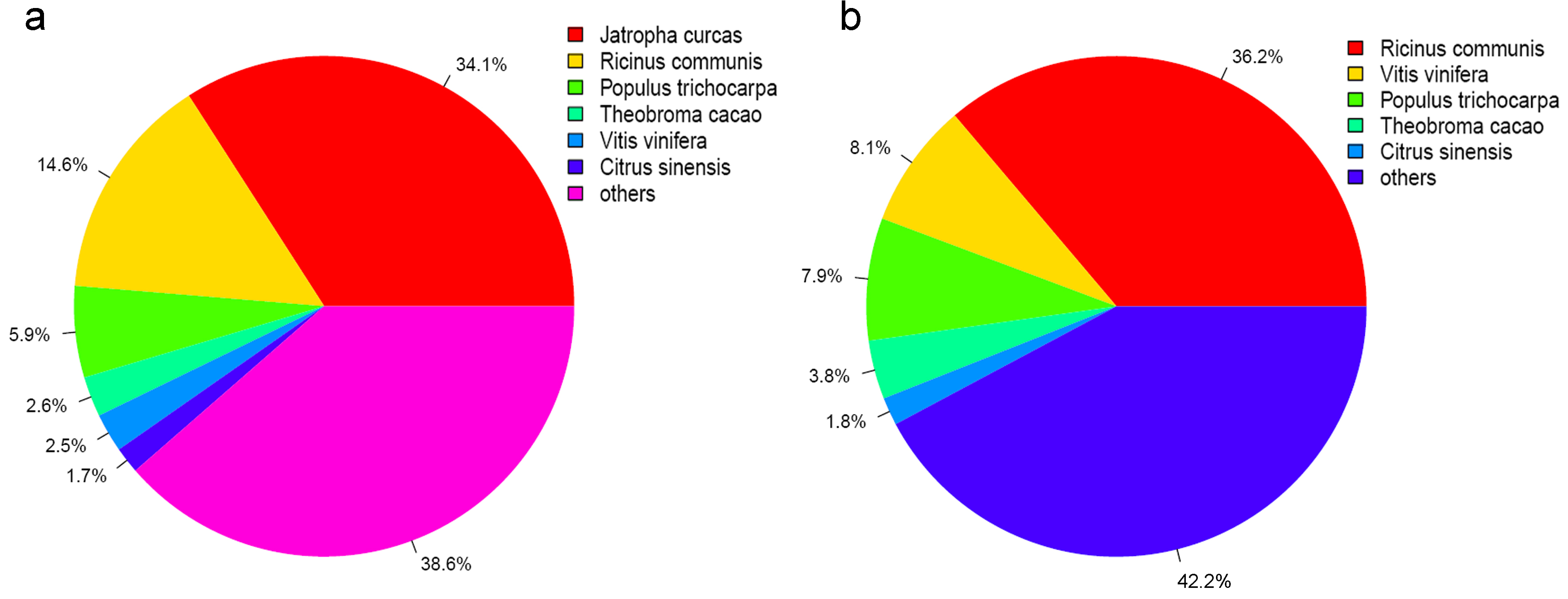

Supplement: Supplemental Figure S1 — The top BLAST species hits with homology to V. fordii (A) and V. morntana (B) according to the transcript annotation. [file Image1.JPEG]

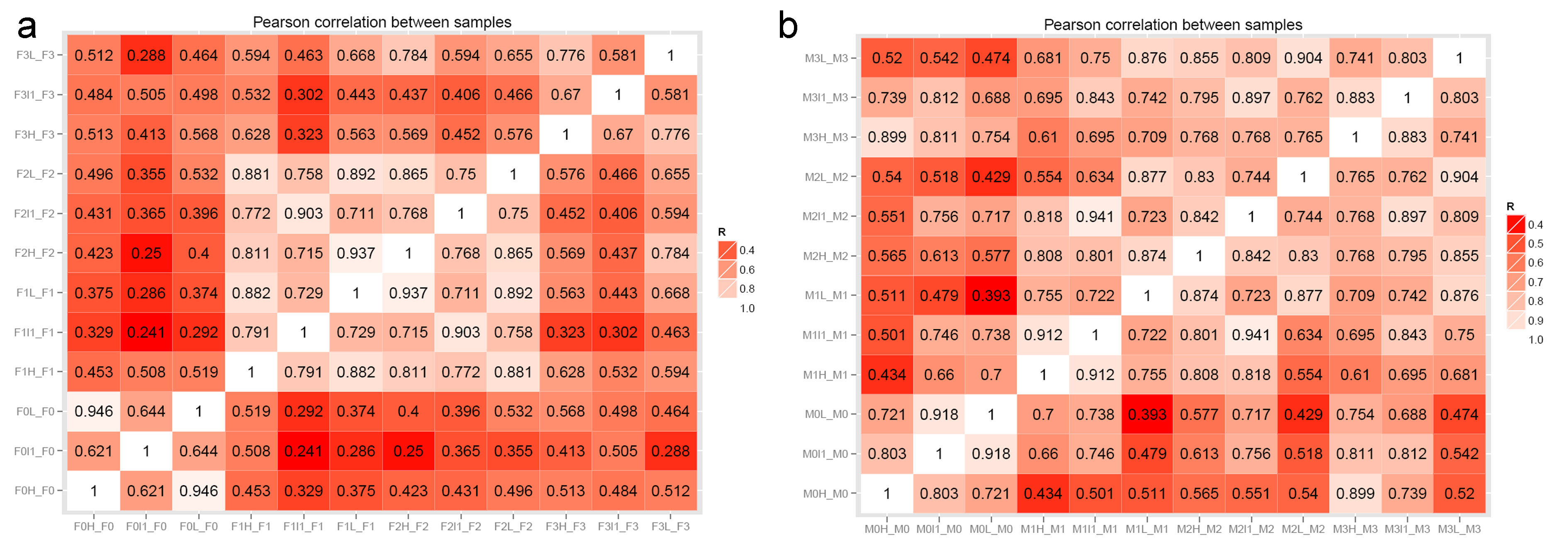

Supplement: Supplemental Figure S2 — Pearson's correlation heat map of the unigenes (FPKM) of all the samples (n = 3) in V. fordii and V. montana. F, V. fordii; M, V. Montana. [file Image2.JPEG]

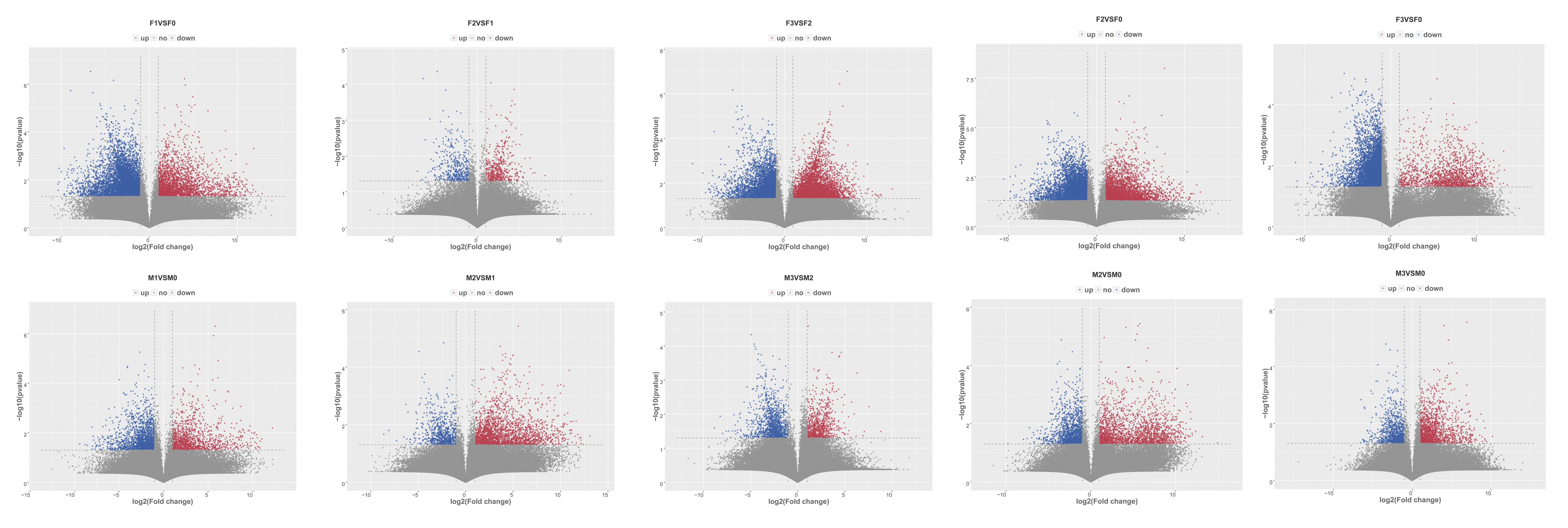

Supplement: Supplemental Figure S3 — A volcano plot using a fold change difference for differential expressed genes in V. fordii and V. montana. [file Image3.JPEG]

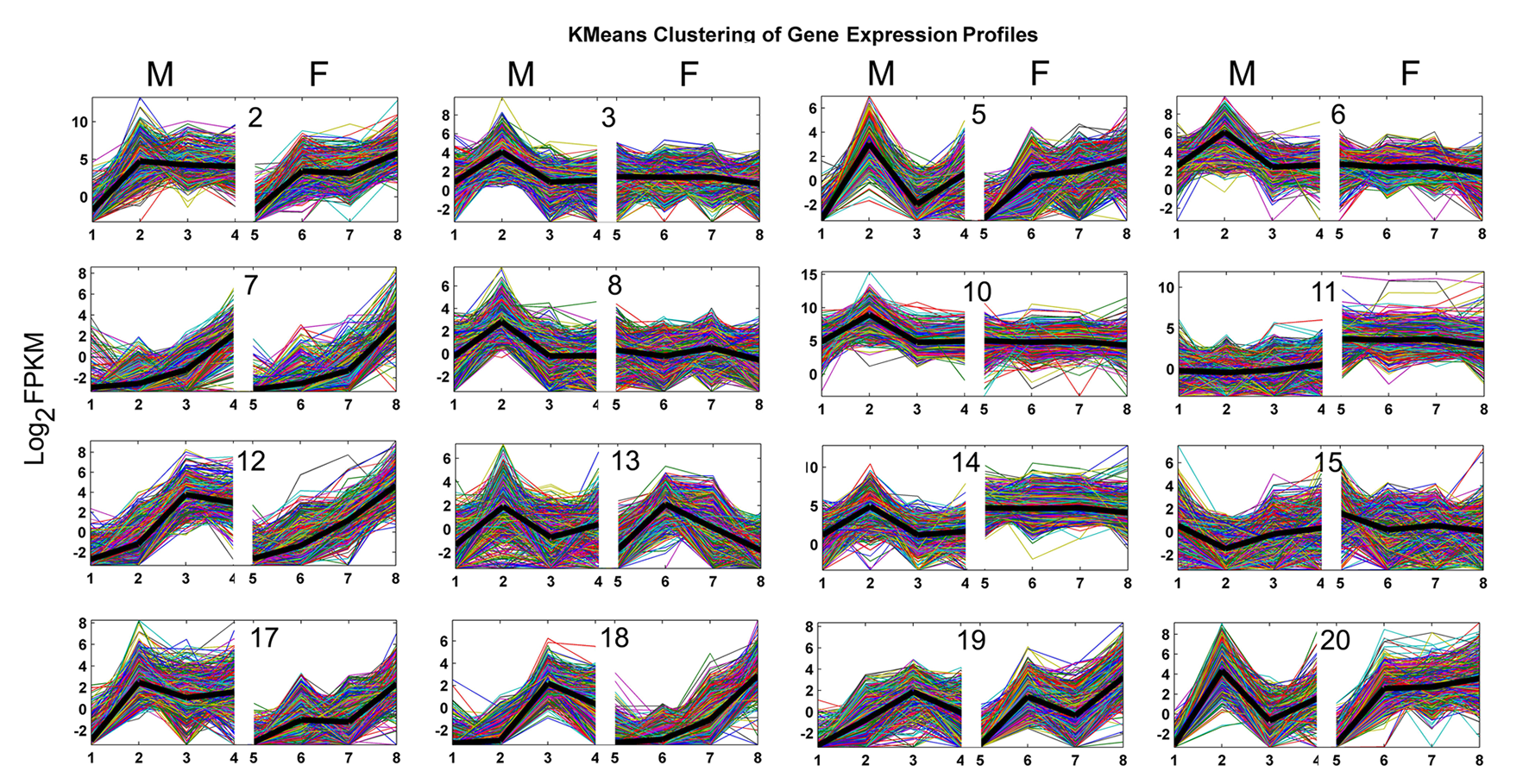

Supplement: Supplemental Figure S4 — A high proportion of orthologous genes exhibited similar expression patterns using k-means clustering of one-to-one orthologous unigene pairs according to the gene expression profiles in V. montana (M) and V. fordii (F) infected with the pathogen F. oxysporum. The differential expression modes were classified into 20 clustersin V. montana, and the corresponding orthologous genes in V. fordii were analyzed for their expression modes (the other four clusters expressed significantly different were shown in Figure 3). [file Image4.TIF]
