# Supplementary material for: Comparative Transcriptomics Atlases Reveals Different Gene Expression Pattern Related to Fusarium Wilt Disease Resistance and Susceptibility in Two Vernicia Species
Source: Front Plant Sci. 2016 Dec 27;7:1974. doi: 10.3389/fpls.2016.01974 (PMC5186792; doi:10.3389/fpls.2016.01974)
Supplement: Supplemental Figure S5 — Verification of the expression profiles of 21 pairs of unigenes by real-time quantitative RT-PCR. X-axis represented the different stage of F. oxysporum infection. 0, the stage before infection; 1, the early stage of infection with F. oxysporum; 2; the middle stage of infection with F. oxysporum; 3, the late stage of infection with F. oxysporum. The left Y-axis represented the expression level (2−ΔΔCT value) of genes. The right Y-axis represented the expression level of genes according to the FPKM value. The blue solid line represented the expression level (2−ΔΔCT) of genes in V. montana (M) using qPCR; the blue dotted line represented the expression level of genes in V. montana (M) according to the FPKM value; the red real line represented the expression level (2−ΔΔCT) of genes in V. fordii (F) using qPCR; the red dotted line represented the expression level of genes in V. fordii (F) according to the FPKM value. [file Image5.PDF]

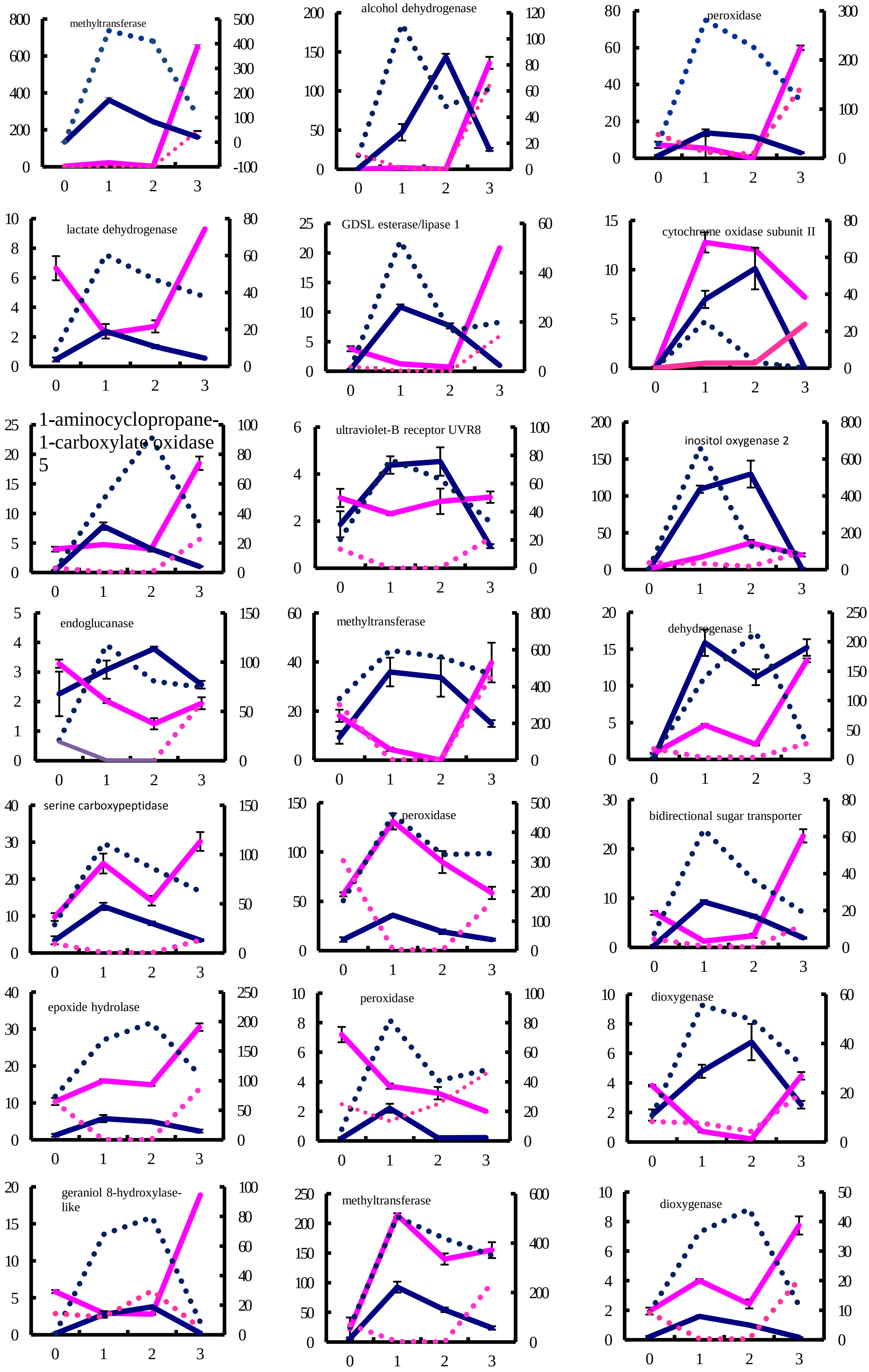
methyltransferase
alcohol dehydrogenase
peroxidase
lactate dehydrogenase
GDSL esterase/lipase 1
cytochrome oxidase subunit II
1-aminocyclopropane-1-carboxylate oxidase 5
ultraviolet-B receptor UVR8
inositol oxygenase 2
endoglucanase
methyltransferase
dehydrogenase 1
serine carboxypeptidase
peroxidase
bidirectional sugar transporter
epoxide hydrolase
peroxidase
dioxygenase
geraniol 8-hydroxylase-like
methyltransferase
dioxygenase

Supplemental Figure S5. Verification of the expression profiles of 21 pairs of unigenes in *V. fordii* and *V. montana* by real-time quantitative RT-PCR. The blue solid line represented the expression level ($2^{-\Delta\Delta CT}$) of genes in *V. montana* using qPCR; the blue dotted line represented the expression level of genes in *V. montana* (M) according to the FPKM value; the red real line represented the expression level ($2^{-\Delta\Delta CT}$) of genes in *V. fordii* (F) using qPCR; the red dotted line represented the expression level of genes in *V. fordii* (F) according to the FPKM value.
