# Supplementary material for: Comparative Transcriptomics Atlases Reveals Different Gene Expression Pattern Related to Fusarium Wilt Disease Resistance and Susceptibility in Two Vernicia Species
Source: Front Plant Sci. 2016 Dec 27;7:1974. doi: 10.3389/fpls.2016.01974 (PMC5186792; doi:10.3389/fpls.2016.01974)
Supplement: Supplemental Figure S6 — Comparative expression patterns of pairs of LRR-RLK genes between susceptible V. fordii and resistant V. montana underlying the response to attack by F. oxysporum. [file Image6.PDF]

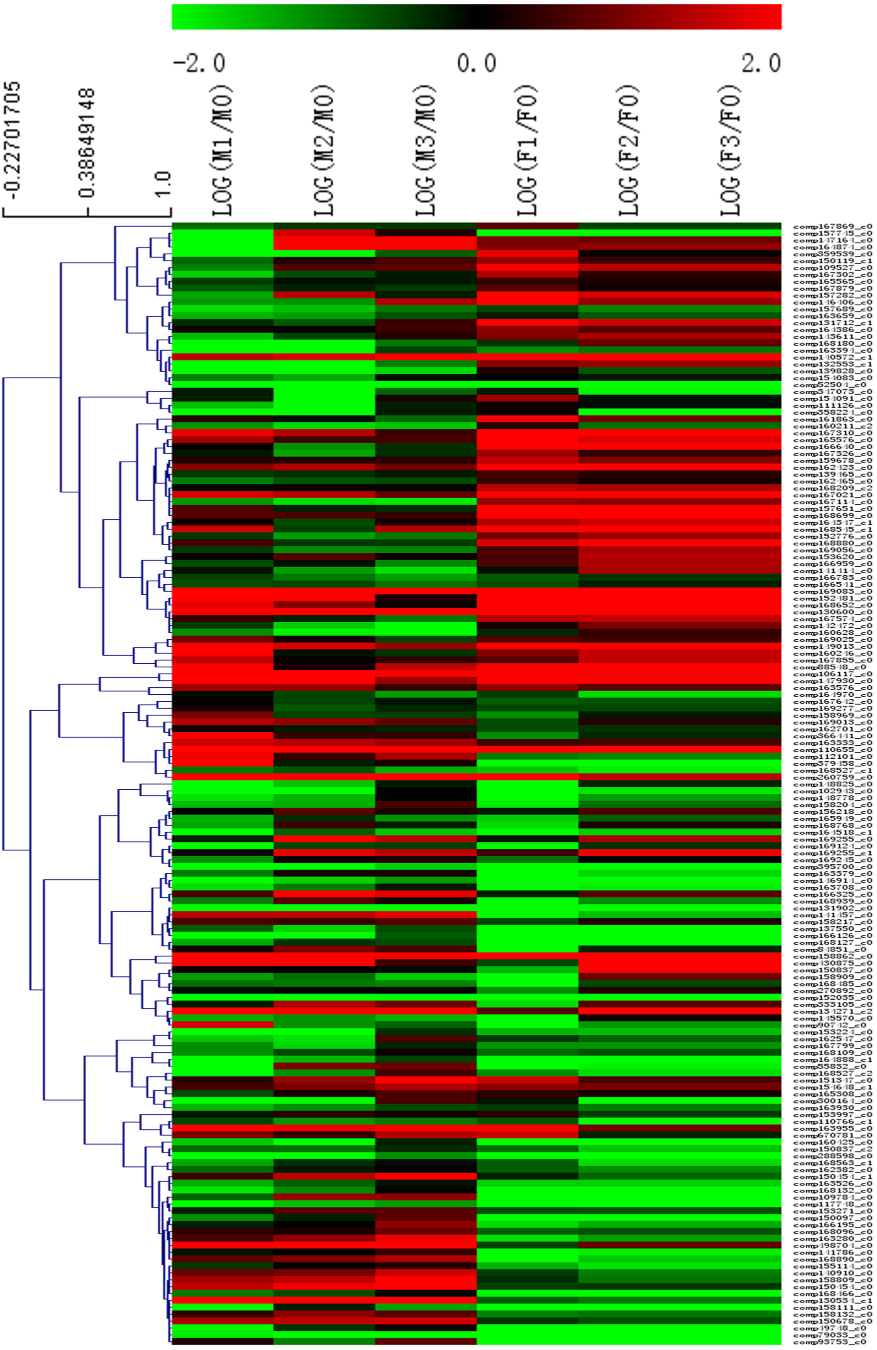

-2.0
0.0
2.0
-0.22701705
0.38649148
1.0
LOG(M1/M0)
LOG(M2/M0)
LOG(M3/M0)
LOG(F1/F0)
LOG(F2/F0)
LOG(F3/F0)

Supplemental Figure S6. Comparative expression patterns of pairs of LRR-RLK genes between susceptible *V. fordii* and resistant *V. montana* underlying the response to attack by *F. oxysporum*.
