# Supplementary material for: Comparative Transcriptomics Atlases Reveals Different Gene Expression Pattern Related to Fusarium Wilt Disease Resistance and Susceptibility in Two Vernicia Species
Source: Front Plant Sci. 2016 Dec 27;7:1974. doi: 10.3389/fpls.2016.01974 (PMC5186792; doi:10.3389/fpls.2016.01974)
Supplement: Supplemental Table S1 — Primer sequences of genes amplification and qPCR in the study. [file Table1.DOCX]

Supplemental Table S1 Primer sequences of genes amplification and qPCR in the study.

| Primer names | Primer sequences (5'-3') | Annealing  temperature | Amplicons length(bp) |
| --- | --- | --- | --- |
| *ITS(*internal transcribed spacer*)* | TCCGTAGGTGAACCTGCGG / TCCTCCGCTTATTGATATGC | 55°C | 500 |
| *EF-1a(translation elongation factor alpha)* | ATGGGTAAGGAAGACAAGAC / GGAAGTACCAGTGATCATGTT | 55°C | 500 |
| *Cytochrome P450* | ACTCCATAGAAATCGCAAACT / CGGCAGATGAAACTACAAG | 80.8 | 179 |
| *PR-4-like* | TTTCTCATAGGTAGGGGCAAT / GAAGGCGGTCCATCCATATT | 84.6 | 171 |
| *chitinase* | TCCTGGCAAGCAGTATTACGG / CGAGGGCTTTGGAGATTGTG | 83.4 | 184 |
| *EREBP-like factor* | TTTAGGATGCGTGGCTCTAAG / CGCATTCCGATTCTGATTGTG | 82.5 | 178 |
| *LRR RLK CLAVATA3* | TATGGTCTAATGGCTAATCTG / GCCTTCGGTGATATTCA | 77.9 | 193 |
| *osmotin* | ACCCAACTCCGGCTTC / TGTAGCGCAGTGGAATTGACC | 88.1 | 154 |
| *salicylic acid-binding protein 2-like* | CCGGGTCCATCCTACAAT / CCAAAGTTGTTGCCAATGTCC | 80.9 | 196 |
| *receptor-like serine/threonine-protein kinase* | TTCTTCTGGAGATCCCATTT / GTAAACCATCCTTTGCCTC | 52.15 | 151 |
| *alcohol dehydrogenase* | AGTGGAATAGGCGAAAG / TCACATGTTACATCGCAATGA | 78.9 | 152 |
| *methyltransferase* | CACCATTTGTAGGCGTAT / GATATATAAGTTTCGGATGGA | 75.7 | 152 |
| *alcohol dehydrogenase 1* | CTAGCACAGCTGGTCAGGTTA / TGATTGTCCCTTAGCTTCC | 85.2 | 179 |
| *peroxidase* | CCAGCACCCACTTCTAACA / GATCTTGGGTTGGATATAGAC | 85.2 | 151 |
| *lactate dehydrogenase* | CAGATCCCAGGCGAGTCTAGG / CCCGATAACCCGATTGGATG | 83.7 | 183 |
| *GDSL esterase/lipase 1* | AAAGGAGAAACTAGGCGATGA / GCCAATAACCATTCCGACGAA | 80.6 | 157 |
| *cytochrome oxidase subunit II* | GCTAAAGCTCACCCGTTA / GGGCAGCCTACTACTTAAGAT | 79.2 | 160 |
| *1-aminocyclopropane-1-carboxylate oxidase 5* | GAGAACTTGGGTTTCGATTAC / GCATTTGGGTCAGTATGTCC | 81.1 | 172 |
| *ultraviolet-B receptor UVR8* | TTTGCGGCCATCGTTAATCAT / GAGGGCGAGAGTATGCGATTC | 84.7 | 198 |
| *ultraviolet-B receptor UVR8* | TTCTGGTGCCCAAATCTACA / CCCATATTCTTCTCGCATCTT | 81 | 191 |
| *Actin7a* | CGATGAAGCACAGTCCAAAAG/GTTGAGAGGAGCCTCAGTG | 58.8 | 170 |
