# Supplementary material for: Comparative Transcriptomics Atlases Reveals Different Gene Expression Pattern Related to Fusarium Wilt Disease Resistance and Susceptibility in Two Vernicia Species
Source: Front Plant Sci. 2016 Dec 27;7:1974. doi: 10.3389/fpls.2016.01974 (PMC5186792; doi:10.3389/fpls.2016.01974)
Supplement: Supplemental Table S2 — Output statistics of sequencing and mapping stats for each sample stage in V. fordii and V. montana infected with F. oxysporum. [file Table2.DOCX]

Supplementary Table S2 Output statistics of sequencing and mapping stats for each stage samples in *V. fordii* and *V. montana* infencted with *F. oxysporum*

| **Sample** | **Raw Data Reads** | **Raw Data Base(Gb)** | **Valid Data Read** | **Valid Data Base(Mb)** | **Valid%** | **Q20%** | **Q30%** | **GC%** |
| --- | --- | --- | --- | --- | --- | --- | --- | --- |
| F0 | 47271474 | 5.9 | 46604130 | 5.8 | 98.59 | 95.80 | 91.94 | 42.65 |
| F0 | 67015042 | 8.4 | 66515356 | 8.3 | 99.25 | 100 | 100 | 44.30 |
| F0 | 29721888 | 3.7 | 29272386 | 3.7 | 98.49 | 94.95 | 90.39 | 43.59 |
| F1 | 31706024 | 3.0 | 31347672 | 3.9 | 98.87 | 95.48 | 91.22 | 44.04 |
| F1 | 63944758 | 8.0 | 63432696 | 7.9 | 99.20 | 100 | 100 | 44.18 |
| F1 | 29973398 | 3.7 | 29620658 | 3.7 | 98.82 | 95.39 | 91.10 | 42.61 |
| F2 | 30190630 | 3.8 | 29482698 | 3.7 | 97.66 | 95.88 | 92.04 | 42.69 |
| F2 | 64798458 | 8.1 | 64285262 | 8.0 | 99.21 | 100 | 100 | 44.41 |
| F2 | 29414036 | 3.7 | 28992470 | 3.6 | 98.57 | 94.79 | 90.03 | 43.20 |
| F3 | 29638854 | 3.7 | 29419992 | 3.7 | 99.26 | 95.06 | 90.48 | 45.47 |
| F3 | 64607354 | 8.1 | 64236976 | 8.0 | 99.43 | 100 | 100 | 45.95 |
| F3 | 33613298 | 4.2 | 33211792 | 4.2 | 98.81 | 94.77 | 90.11 | 44.37 |
| M0 | 42908608 | 5.4 | 42329318 | 5.3 | 98.65 | 95.63 | 91.48 | 42.63 |
| M0 | 67365500 | 8.4 | 66859072 | 8.4 | 99.25 | 100.00 | 100.00 | 43.98 |
| M0 | 41658080 | 5.2 | 41093632 | 5.1 | 98.65 | 95.08 | 90.63 | 44.22 |
| M1 | 41020054 | 5.2 | 39967224 | 5.0 | 97.43 | 96.37 | 93.10 | 43.60 |
| M1 | 66257284 | 8.3 | 65782492 | 8.2 | 99.28 | 100.00 | 100.00 | 44.87 |
| M1 | 39510126 | 4.9 | 39077922 | 4.9 | 98.91 | 95.35 | 91.08 | 42.84 |
| M2 | 30866850 | 3.9 | 30558196 | 3.8 | 99.00 | 94.76 | 89.87 | 43.32 |
| M2 | 66689614 | 8.4 | 66242736 | 8.3 | 99.33 | 100.00 | 100.00 | 45.13 |
| M2 | 32928438 | 4.1 | 32451888 | 4.1 | 98.55 | 95.97 | 92.26 | 43.03 |
| M3 | 37307274 | 4.7 | 36548912 | 4.6 | 97.97 | 95.96 | 92.27 | 43.30 |
| M3 | 64703606 | 8.1 | 64240712 | 8.0 | 99.28 | 100.00 | 100.00 | 44.11 |
| M3 | 34427796 | 4.3 | 34029072 | 4.3 | 98.84 | 95.14 | 90.69 | 44.22 |

Q20: percentage is the proportion of nucleotides with a quality value ≥20 in reads.

Q30: percentage is the proportion of nucleotides with a quality value ≥30 in reads.
