# Supplementary material for: Comparative Transcriptomics Atlases Reveals Different Gene Expression Pattern Related to Fusarium Wilt Disease Resistance and Susceptibility in Two Vernicia Species
Source: Front Plant Sci. 2016 Dec 27;7:1974. doi: 10.3389/fpls.2016.01974 (PMC5186792; doi:10.3389/fpls.2016.01974)
Supplement: Supplemental Table S6 — The molecular evolution of orthologous genes between V. fordii and V. montana under accelerated evolution with ratios of Ka/Ks>1 and p < 0.05. [file Table6.DOCX]

Supplemental Table S6 Annotation to 92 sequences under accelerated evolution with rations of Ka/Ks>1 and pvalue < 0.05

| **Sequence F:M** | **Ka/Ks** | **P-Value**  **(Fisher)** | **Annotation** | **Divergence-Time** |
| --- | --- | --- | --- | --- |
| comp196617_c0_No.1:comp166152_c0_No.1 | 4.03 | 0.0000 | Zn2+-exporting ATPase | 4.42 |
| comp176763_c0_No.1:comp129816_c0_No.1 | 6241.45 | 0.0172 | xyloglucan:xyloglucosyl transferase | 0.02 |
| comp195926_c0_No.2:comp164899_c0_No.1 | 1.84 | 0.0004 | WD repeat-containing protein 21A | 4.35 |
| comp83908_c0_No.1:comp672076_c0_No.1 | 13.29 | 0.0061 | ubiquitin-conjugating enzyme E2 A | 0.20 |
| comp194817_c0_No.1:comp168051_c0_No.2 | 3.21 | 0.0010 | tRNA nucleotidyltransferase (CCA-adding enzyme) | 4.26 |
| comp193924_c0_No.2:comp163254_c0_No.2 | 3.22 | 0.0000 | transducin (beta)-like 1 | 4.56 |
| comp188287_c0_No.1:comp166863_c0_No.1 | 2.31 | 0.0000 | threonine synthase | 4.58 |
| comp196311_c0_No.2:comp165648_c0_No.1 | 2.91 | 0.0150 | thiol oxidase | 0.06 |
| comp195500_c0_No.1:comp161227_c0_No.2 | 3.78 | 0.0000 | symplekin | 4.73 |
| comp193360_c0_No.1:comp154388_c0_No.1 | 1.30 | 0.0176 | SWI/SNF-related matrix-associated actin-dependent regulator of chromatin subfamily A member 2/4 | 2.39 |
| comp194066_c0_No.1:comp165933_c0_No.1 | 2.35 | 0.0000 | SWI/SNF related-matrix-associated actin-dependent regulator of chromatin subfamily C | 2.27 |
| comp183520_c1_No.1:comp167620_c0_No.1 | 3.39 | 0.0000 | steroid 23-alpha-hydroxylase | 3.57 |
| comp187259_c0_No.1:comp145889_c0_No.1 | 2.61 | 0.0414 | STE20-like kinase | 4.61 |
| comp195148_c0_No.2:comp165414_c2_No.2 | 2.81 | 0.0000 | spore coat protein A | 4.25 |
| comp85710_c0_No.1:comp911077_c0_No.1 | 2.61 | 0.0089 | small subunit ribosomal protein S6e | 3.23 |
| comp106417_c0_No.1:comp131049_c1_No.1 | 2.72 | 0.0271 | small subunit ribosomal protein S5e | 0.17 |
| comp191598_c0_No.1:comp167827_c0_No.1 | 3.01 | 0.0331 | sinapoylglucose-choline O-sinapoyltransferase | 0.03 |
| comp191152_c0_No.1:comp162050_c0_No.1 | 2.37 | 0.0001 | ribosomal RNA small subunit methyltransferase F | 2.88 |
| comp195245_c0_No.1:comp158762_c0_No.1 | 1.82 | 0.0000 | ribosomal large subunit pseudouridine synthase B | 2.34 |
| comp187696_c0_No.1:comp160824_c0_No.1 | 3.16 | 0.0001 | ribose 5-phosphate isomerase A | 3.88 |
| comp367706_c0_No.1:comp469594_c0_No.1 | 4.28 | 0.0000 | receptor-interacting serine/threonine-protein kinase 4 | 3.21 |
| comp191840_c0_No.1:comp163732_c1_No.1 | 1.88 | 0.0008 | protoheme IX farnesyltransferase | 1.69 |
| comp193453_c0_No.1:comp169083_c0_No.1 | 2.40 | 0.0117 | protein-serine/threonine kinase | 0.07 |
| comp193285_c0_No.1:comp165565_c0_No.1 | 3.06 | 0.0000 | protein-serine/threonine kinase | 4.50 |
| comp191843_c0_No.1:comp160690_c0_No.1 | 2.50 | 0.0000 | protein-serine/threonine kinase | 4.67 |
| comp189711_c0_No.1:comp166409_c1_No.2 | 3.34 | 0.0000 | protein phosphatase | 4.36 |
| comp186968_c0_No.1:comp147752_c0_No.1 | 6.80 | 0.0000 | prephenate dehydratase | 3.64 |
| comp195806_c0_No.1:comp168107_c0_No.1 | 1.68 | 0.0225 | pre-mRNA-processing factor 4 | 2.44 |
| comp176279_c0_No.1:comp127850_c0_No.1 | 5.82 | 0.0000 | pre-mRNA-processing factor 4 | 4.03 |
| comp195288_c0_No.1:comp168588_c0_No.1 | 1.68 | 0.0055 | phosphoribosylaminoimidazole carboxylase | 4.73 |
| comp195715_c0_No.1:comp167792_c0_No.2 | 3.40 | 0.0000 | phospholipase D | 4.68 |
| comp194028_c0_No.1:comp151041_c0_No.1 | 4.38 | 0.0040 | phospholipase C, delta | 0.04 |
| comp193059_c0_No.1:comp158191_c1_No.1 | 12.42 | 0.0000 | peroxidase | 0.06 |
| comp190475_c0_No.1:comp158191_c0_No.1 | 24.72 | 0.0000 | peroxidase | 0.05 |
| comp195256_c0_No.1:comp167840_c1_No.1 | 1.13 | 0.0000 | nucleolin | 3.54 |
| comp190950_c0_No.2:comp167080_c0_No.2 | 1.61 | 0.0000 | nucleolin | 2.49 |
| comp195963_c0_No.1:comp167974_c0_No.2 | 2.98 | 0.0000 | non-specific serine/threonine protein kinase | 3.02 |
| comp194318_c1_No.1:comp165157_c0_No.1 | 2.26 | 0.0123 | nitrite reductase (NO-forming) | 0.06 |
| comp196472_c0_No.1:comp166332_c1_No.2 | 2.32 | 0.0000 | NIMA (never in mitosis gene a)-related kinase | 4.38 |
| comp191368_c0_No.1:comp166025_c0_No.1 | 3.30 | 0.0000 | myosin V | 5.02 |
| comp186984_c0_No.1:comp163407_c0_No.1 | 3.28 | 0.0000 | myo-inositol-1(or 4)-monophosphatase | 3.84 |
| comp195584_c2_No.1:comp161615_c1_No.1 | 1.07 | 0.0000 | molecular chaperone HtpG | 4.45 |
| comp196012_c0_No.1:comp168289_c0_No.2 | 3.04 | 0.0000 | major histocompatibility complex, class I | 3.86 |
| comp190815_c0_No.1:comp151299_c0_No.1 | 2.93 | 0.0000 | large subunit ribosomal protein LP2 | 3.12 |
| comp282781_c0_No.1:comp420718_c0_No.1 | 5.35 | 0.0002 | large subunit ribosomal protein L7e | 0.17 |
| comp130880_c0_No.1:comp154765_c3_No.1 | 4.27 | 0.0197 | large subunit ribosomal protein L3e | 0.10 |
| comp184900_c0_No.1:comp162459_c0_No.1 | 2.95 | 0.0008 | large subunit ribosomal protein L29e | 2.95 |
| comp194037_c0_No.2:comp155857_c0_No.1 | 1.71 | 0.0000 | KUP system potassium uptake protein | 4.96 |
| comp185106_c2_No.1:comp155611_c0_No.1 | 2.39 | 0.0000 | kinesin family member 5 | 4.43 |
| comp194327_c0_No.1:comp165463_c0_No.2 | 1.50 | 0.0165 | kinesin family member 15 | 2.03 |
| comp179009_c0_No.1:comp159604_c0_No.1 | 2.13 | 0.0057 | kinesin family member 11 | 3.09 |
| comp192682_c0_No.2:comp165716_c0_No.1 | 2.98 | 0.0001 | jumonji domain-containing protein 1 | 4.85 |
| comp188281_c1_No.1:comp149502_c0_No.1 | 6.32 | 0.0000 | jumonji domain-containing protein 1 | 3.86 |
| comp184178_c0_No.1:comp156633_c0_No.1 | 1.19 | 0.0039 | isocitrate dehydrogenase (NAD+) | 2.99 |
| comp421835_c0_No.1:comp91809_c0_No.1 | 5.67 | 0.0000 | inorganic pyrophosphatase | 2.75 |
| comp182303_c0_No.1:comp163735_c0_No.1 | 3.81 | 0.0000 | hydroquinone glucosyltransferase | 4.27 |
| comp180733_c0_No.1:comp64723_c0_No.1 | 3.12 | 0.0000 | homogentisate solanesyltransferase | 3.96 |
| comp183115_c5_No.1:comp167733_c1_No.1 | 2.59 | 0.0456 | heat shock protein 90kDa beta | 4.07 |
| comp178771_c3_No.1:comp152388_c1_No.1 | 3.88 | 0.0000 | glutamate receptor, ionotropic, other eukaryote | 3.50 |
| comp190840_c1_No.1:comp165489_c0_No.1 | 3.60 | 0.0000 | fused | 4.35 |
| comp186107_c1_No.1:comp169084_c0_No.1 | 2.80 | 0.0000 | fructose-bisphosphate aldolase, class I | 0.15 |
| comp194790_c0_No.1:comp167734_c0_No.1 | 3.34 | 0.0053 | FAD synthetase | 0.04 |
| comp192655_c0_No.1:comp166225_c0_No.2 | 1.74 | 0.0000 | exopolyphosphatase | 5.08 |
| comp192642_c0_No.1:comp164758_c0_No.1 | 1.28 | 0.0013 | EREBP-like factor | 2.80 |
| comp179159_c0_No.1:comp139704_c0_No.2 | 4.87 | 0.0000 | EREBP-like factor | 3.74 |
| comp195153_c0_No.1:comp158353_c0_No.1 | 5.06 | 0.0000 | ent-kaurenoic acid hydroxylase | 3.95 |
| comp193383_c0_No.1:comp166490_c0_No.2 | 2.74 | 0.0000 | enhancer of polycomb-like protein | 4.30 |
| comp187589_c0_No.1:comp166087_c0_No.2 | 2.35 | 0.0000 | E3 ubiquitin-protein ligase UHRF1 | 4.41 |
| comp191677_c0_No.1:comp151367_c0_No.1 | 2.42 | 0.0148 | dynamin GTPase | 0.22 |
| comp195250_c0_No.1:comp168955_c1_No.1 | 4.62 | 0.0000 | dual-specificity tyrosine-(Y)-phosphorylation regulated kinase | 3.65 |
| comp193381_c1_No.1:comp166851_c0_No.2 | 3.31 | 0.0000 | dual-specificity tyrosine-(Y)-phosphorylation regulated kinase | 4.92 |
| comp189668_c0_No.2:comp156137_c0_No.1 | 3.01 | 0.0001 | DnaJ homolog, subfamily A, member 2 | 4.36 |
| comp192304_c0_No.1:comp168554_c0_No.2 | 3.12 | 0.0000 | DNA-directed RNA polymerase II subunit A | 4.57 |
| comp186182_c1_No.1:comp158244_c0_No.1 | 3.48 | 0.0000 | DNA topoisomerase III | 4.09 |
| comp184059_c0_No.1:comp156615_c1_No.2 | 3.25 | 0.0015 | DNA excision repair protein ERCC-1 | 4.01 |
| comp186498_c0_No.1:comp146601_c0_No.1 | 2.26 | 0.0000 | death-associated protein kinase | 2.36 |
| comp196029_c0_No.1:comp167009_c0_No.1 | 3.54 | 0.0000 | chromodomain-helicase-DNA-binding protein 1 | 5.10 |
| comp189240_c0_No.2:comp161815_c0_No.2 | 2.10 | 0.0008 | chromobox protein 4 | 2.58 |
| comp189492_c0_No.1:comp163555_c0_No.1 | 1.56 | 0.0000 | carboxypeptidase | 2.72 |
| comp186366_c1_No.1:comp152655_c0_No.1 | 4.51 | 0.0000 | bromodomain-containing protein 4 | 3.17 |
| comp191301_c0_No.2:comp165499_c0_No.1 | 3.97 | 0.0000 | beta-glucosidase | 3.99 |
| comp194706_c0_No.1:comp168334_c0_No.2 | 1.74 | 0.0000 | argonaute | 3.29 |
| comp191110_c0_No.1:comp165270_c1_No.2 | 2.13 | 0.0062 | alanine transaminase | 2.88 |
| comp180444_c1_No.1:comp158625_c0_No.1 | 2.78 | 0.0000 | adenylate kinase | 3.89 |
| comp193269_c0_No.1:comp169123_c1_No.1 | 2.16 | 0.0001 | adenosinetriphosphatase | 0.07 |
| comp196246_c0_No.1:comp168803_c0_No.2 | 2.51 | 0.0000 | adenosinetriphosphatase | 3.12 |
| comp196599_c0_No.1:comp169320_c0_No.1 | 2.46 | 0.0358 | acetyl-CoA carboxylase / biotin carboxylase | 0.01 |
| comp186750_c0_No.1:comp155560_c0_No.2 | 3.05 | 0.0000 | aarF domain-containing kinase | 4.35 |
| comp194041_c0_No.1:comp161011_c0_No.1 | 2.84 | 0.0000 | 6-phosphofructokinase | 3.97 |
| comp188343_c0_No.1:comp165086_c0_No.1 | 1.26 | 0.0497 | 3-hydroxyisobutyrate dehydrogenase | 2.45 |
| comp187669_c0_No.1:comp167803_c0_No.2 | 4.28 | 0.0000 | 24-methylenesterol C-methyltransferase | 3.59 |
| comp193089_c0_No.1:comp156461_c0_No.1 | 2.46 | 0.0008 | (R)-3-amino-2-methylpropionate-pyruvate transaminase | 4.48 |
